# Unraveling the Pathogenesis of Post‐Stroke Depression in a Hemorrhagic Mouse Model through Frontal Lobe Circuitry and JAK‐STAT Signaling

**DOI:** 10.1002/advs.202402152

**Published:** 2024-07-01

**Authors:** Yingqing Wu, Jia Deng, Jinsong Ma, Yujie Chen, Ning Hu, Shilei Hao, Bochu Wang

**Affiliations:** ^1^ Key Laboratory of Biorheological Science and Technology, Ministry of Education, College of Bioengineering Chongqing University Chongqing 400030 China; ^2^ College of Environment and Resources Chongqing Technology and Business University Chongqing 400030 China; ^3^ Department of Neurosurgery and State Key Laboratory of Trauma, Burn and Combined Injury, Southwest Hospital Third Military Medical University (Army Medical University) Chongqing 400038 China

**Keywords:** circuitry connectivity, electrophysiological alterations, frontal lobe hemorrhage, hematoma volumes, post‐stroke depression

## Abstract

Post‐stroke depression is a common complication that imposes significant burdens and challenges on patients. The occurrence of depression is often associated with frontal lobe hemorrhage, however, current understanding of the underlying mechanisms remains limited. Here, the pathogenic mechanisms associated with the circuitry connectivity, electrophysiological alterations, and molecular characteristics are investigated related to the frontal lobe in adult male mice following unilateral injection of blood in the medial prefrontal cortex (mPFC). It is demonstrated that depression is a specific neurological complication in the unilateral hematoma model of the mPFC, and the ventral tegmental area (VTA) shows a higher percentage of connectivity disruption compared to the lateral habenula (LHb) and striatum (STR). Additionally, long‐range projections originating from the frontal lobe demonstrate higher damage percentages within the connections between each region and the mPFC. mPFC neurons reveal reduced neuronal excitability and altered synaptic communication. Furthermore, transcriptomic analysis identifies the involvement of the Janus Kinase‐Signal Transducer and Activator of Transcription (JAK‐STAT) signaling pathway, and targeting the JAK‐STAT pathway significantly alleviates the severity of depressive symptoms. These findings improve the understanding of post‐hemorrhagic depression and may guide the development of efficient treatments.

## Graphical abstract

Researchers investigate post‐stroke depression in mice with frontal lobe hematoma, finding disrupted connectivity in the ventral tegmental area and damage to long‐range projections from the frontal lobe. Reduced neuronal excitability, altered synaptic communication and JAK‐STAT signaling pathway activation have also been identified. These findings enhance understanding and treatment possibilities for post‐hemorrhagic depression.

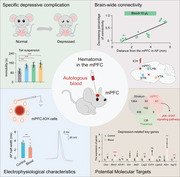

## 1 Introduction

Post‐stroke depression is a common complication following a stroke, affecting an estimated one‐third to half of stroke survivors.^[^ ^1^ ^]^ Stroke can be categorized into two types: ischemic stroke (80%) and hemorrhagic stroke (20%).^[^ ^2^ ^]^ Among them, survivors of intracerebral hemorrhage (ICH) have a significantly high risk of post‐stroke depression, with reported occurrence rates as high as 60%.^[^ ^3^ ^]^ Post‐stroke depression can exacerbate disability, worsen cognitive function, increase mortality rates, and impair recovery.^[^ ^4^ ^]^ Therefore, understanding the underlying mechanisms is crucial to comprehend the occurrence and development of depressive symptoms. This understanding provides a scientific foundation for early identification, personalized treatment approaches, and the development of rehabilitation plans, ultimately improving the psychological well‐being and quality of life of stroke survivors. Currently, the pathogenesis of post‐stroke depression involves four main factors: biological factors, neural circuitry alterations, genetic factors, and psychosocial factors.^[^ ^5^ ^]^ Biological factors primarily involve imbalances in neurotransmitters, such as serotonin, dopamine, and norepinephrine.^[^ ^6^ ^]^ Neural circuitry alterations manifest as abnormalities in circuits responsible for emotion regulation, cognitive control, and reward systems.^[^ ^7^ ^]^ Genetic factors are associated with genes involved in the neurotransmitter system and stress responses (e.g., *SLC6A4*, *VDR*, *FKBP5*, *CRHR1*).^[^ ^8^ ^]^ In the case of ICH, the primary brain injury caused by the bleeding itself triggers biological responses, disrupts neural circuitry, and may have underlying genetic predispositions,^[^ ^9^ ^]^ all of which contribute to the development of post‐stroke depression. However, research on the specific mechanisms underlying post‐stroke depression induced by ICH is currently limited, with most studies focusing on related biochemical markers.^[^ ^10^ ^]^ Exploring the potential mechanisms of post‐stroke depression caused by ICH holds significant clinical importance in improving depressive symptoms, rehabilitation, and the quality of life for affected patients.

The frontal lobe, striatum, and thalamus, are not only commonly affected sites in ICH but also relevant to the pathophysiology of depression.^[^ ^11^ ^]^ The frontal lobe plays a crucial role in cognitive and behavioral functions, including attention, memory, and emotional regulation.^[^ ^12^ ^]^ Clinical reports indicate that up to 70% of patients with frontal lobe hemorrhage develop depression.^[^ ^13^ ^]^ Especially in the case of the medial prefrontal cortex (mPFC), an important subdivision of the frontal lobe, studies on focal ischemic stroke in the left mPFC have demonstrated a strong phenotype of post‐stroke depression.^[^ ^14^ ^]^ Therefore, it is essential to gain a comprehensive understanding of the depressive characteristics and the underlying mechanisms resulting from frontal lobe hemorrhage. Regarding frontal lobe neural circuits, the ventral tegmental area‐medial prefrontal cortex (VTA‐mPFC) circuit is closely associated with emotion regulation and reward systems,^[^ ^15^ ^]^ while the medial prefrontal cortex‐lateral habenular nucleus (mPFC‐LHb) circuit is involved in negative emotion regulation and reward system inhibition,^[^ ^16^ ^]^ and the medial prefrontal cortex‐striatum (mPFC‐STR) circuit is related to emotion regulation and cognitive control.^[^ ^17^ ^]^ Furthermore, studies have found a close relationship between electrophysiological abnormalities and the occurrence and persistence of depression.^[^ ^18^ ^]^ For example, patients with depression may exhibit increased theta and alpha wave activity in the prefrontal cortex, while beta and gamma wave activity may be reduced.^[^ ^19^ ^]^ Additionally, under ischemic conditions, abnormal activation of inflammation‐related molecules and signaling pathways may occur, leading to disturbances in the neurotransmitter system and impairments in emotional regulation, such as the Nuclear Factor kappa B (NF‐κB) signaling pathway.^[^ ^20^ ^]^ Therefore, investigating frontal lobe circuit connections, electrophysiological abnormalities, and molecular characteristics can significantly deepen our understanding of the pathogenesis and pathophysiological processes of depression caused by right frontal lobe hematoma in adult male C57BL6 mice, providing valuable insights for the development of targeted treatment strategies.

In this study, we hypothesize that depression caused by frontal lobe hematoma is based on pathological characteristics involving circuit connections, electrophysiology, and molecular factors. To validate this hypothesis, we employed a mouse model of frontal lobe hematoma and conducted comprehensive investigations using behavioral assays, viral tracing, immunofluorescence, electrophysiology, and transcriptomic sequencing. Our research findings demonstrate that depression is a specific neurological complication of frontal lobe hematoma. Within the depression circuit of the medial prefrontal cortex, a higher percentage of connectivity impairment was observed in the VTA compared to the LHb and STR, and higher percentages of damage were observed in long‐distance projections originating from the frontal lobe. Furthermore, the hematoma also led to decreased neuronal excitability, manifested by prolonged action potential (AP) duration and decay time. Importantly, comparative transcriptomic analysis of different hematoma locations revealed an enrichment of the Janus Kinase‐Signal Transducer and Activator of Transcription (JAK‐STAT) signaling pathway in frontal lobe hematoma and targeted pharmacological intervention of the JAK‐STAT pathway in mice mitigated the severity of depression symptoms. These findings significantly deepen our understanding of the pathological characteristics of depression following frontal lobe hemorrhage and identify potential targets for treating depression caused by frontal lobe hemorrhage.

## 2 Results

### 2.1 Hematoma in the mPFC Region Leads to Depressive Complications in Mice

**Figure 1:**
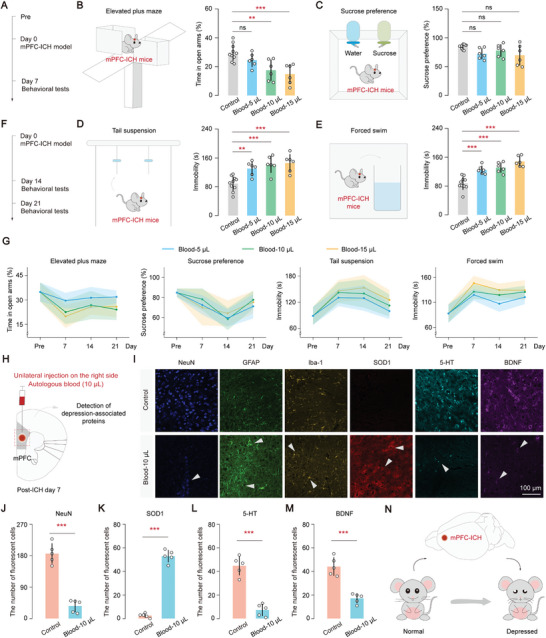
Hematoma in the mPFC region leads to depressive complications in mice. A) Diagram illustrating the measurements of depression‐related behaviors on day 7 following hematoma in the mPFC of mice. B–E) Effect of different hematoma volumes on the elevated plus maze (B), sucrose preference (C), tail suspension (D), and forced swim (E) on post‐ICH day 7. Statistical analysis (one‐way ANOVA and Tukey's multiple comparison test): (B) elevated plus maze, *P* = 0.0003, *P* (Control, Blood 5 µL) = 0.3666, *P* (Control, Blood 10 µL) = 0.0041, *P* (Control, Blood 15 µL) = 0.0005; (C) Sucrose preference, *P* = 0.0555, *P* (Control, Blood 5 µL) = 0.1518, *P* (Control, Blood 10 µL) = 0.6392, *P* (Control, Blood 15 µL) = 0.0637; (D) Tail suspension, *P* < 0.0001, *P* (Control, Blood 5 µL) = 0.0061, *P* (Control, Blood 10 µL) = 0.0005, *P* (Control, Blood 15 µL) = 0.0002; (E) Forced swim, *P* < 0.0001, *P* (Control, Blood 5 µL) = 0.0005, *P* (Control, Blood 10 µL) < 0.0001, *P* (Control, Blood 15 µL) < 0.0001; F) Diagram illustrating the measurements of depression‐related behaviors on day 14 and day 21 following hematoma. G) Trend plot illustrating the impact of various hematoma volumes over time on the elevated plus maze, sucrose preference, tail suspension, and forced swim tests in these groups following hematoma in the mPFC. The periods studied included ICH‐pre, day 7, day 14, and day 21. H) Diagram illustrating the detection of depression‐associated proteins on day 7 following hematoma in the mPFC of mice. I) Changes in protein levels of depression markers, including neuron (NeuN), astrocytes (GFP), microglia (Iba‐1), superoxide dismutase1 (SOD1), 5‐hydroxytryptamine (5‐HT), and brain derived neurotrophic factor (BDNF). Scale bar, 100 µm. J–M) Number of fluorescent cells in different groups. Number of fluorescent cells was analyzed through Image J. Statistical analysis (unpaired t‐tests (parametric tests)): (J) Neuron (NeuN): “Control group”: 186.40, “Blood‐10 µL group”: 36.40. *P* (Control, Blood‐10 µL) < 0.0001; (K) Superoxide dismutase1 (SOD1): “Control group”: 2.60, “Blood‐10 µL group”: 53.00. *P* (Control, Blood‐10 µL) < 0.0001; (L) 5‐hydroxytryptamine (5‐HT): “Control group”: 44.80, “Blood‐10 µL group”: 7.40. *P* (Control, Blood‐10 µL) < 0.0001; (M) Brain derived neurotrophic factor (BDNF): “Control group”: 44.20, “Blood‐10 µL group”: 17.20. *P* (Control, Blood‐10 µL) = 0.0001. N) Hematoma in the mPFC region may lead to the development of neurological complications associated with depression in mice. Data are mean ± sd. In (A–G), control group, *n* = 10 mice; blood group, *n* = 6 mice. In (H‐M), each group, *n* = 5 mice. Not significant (ns), *P* < 0.01(**), and *P* < 0.001(***).

We first examined the effects of mPFC‐ICH on depression‐related behaviors by injecting different volumes of autologous blood (**Figure** **1**A). The time spent in the open arms of the elevated plus maze was significantly reduced in the blood 10 µL and blood 15 µL groups compared to the control group (*P* < 0.01, Figure 1B). Additionally, the results revealed a significant increase in immobility duration during the tail suspension and forced swim tests in the different blood groups compared to the control group on day 7 (*P* < 0.01, Figure 1D,E). However, there were no differences in sucrose preference between the different blood groups and the control group (*P* > 0.05, Figure 1C). This finding suggests that the complex interplay of neural circuits, compensatory mechanisms, and temporal dynamics may contribute to the regulation of sucrose preference.^[^ ^21^ ^]^ The above findings suggest that hematoma in the mPFC can result in neurological complications associated with depression on day 7. Importantly, the severity of depression demonstrated a positive correlation with the hematoma volume, as indicated by various depression‐related indicators (Figure S1A, Supporting Information). Additionally, Cresyl Violet (CV) staining further demonstrates the association between the size of the lesion area and the size of the hematoma (Figure S1B,C, Supporting Information). To better understand the temporal changes in depression following hematoma in the mPFC, a line graph was plotted to illustrate the modifications in depression‐related behaviors on day 7, day 14, and day 21 (Figure 1F,G, Table S1, Supporting Information). Significant disparities were observed in all depression‐related behaviors on both day 7 and day 14, with increased immobility duration in the tail suspension and forced swim tests, reduced time spent in the open arms of the elevated plus maze, and decreased sucrose preference across the various blood groups (Figure 1G; Figure S2A, Supporting Information). However, by day 21, depression had partially been alleviated, as evidenced by increased sucrose preference and decreased immobility duration in the tail suspension test (Figure 1G; Figure S2B, Supporting Information). These findings indicate that depression is a prominent pathological feature in mice with hematoma in the mPFC on both day 7 and day 14.

To evaluate changes in depression‐related markers at the protein level, immunofluorescence techniques were employed following hematoma in the mPFC (Figure 1H,I). Our analysis revealed a significant downregulation of the neuronal marker (NeuN), serotonin (5‐HT), and brain‐derived neurotrophic factor (BDNF) compared to control mice (*P* < 0.001, Figure 1J,L,M). Additionally, a notable upregulation of superoxide dismutase1 (SOD1) was observed in the blood 10 µL group on day 7 compared to the control group (*P* < 0.001, Figure 1K). Furthermore, compared to the control group, the fluorescence intensity of astrocytes (GFAP) and microglia (Iba‐1) was significantly enhanced in the blood 10 µL group (*P* < 0.05), indicating an increased level of activation of GFAP and Iba‐1 near the hematoma (Figure 1I; Figure S2C,D, Supporting Information). These results provide compelling evidence that hematoma in the mPFC contributes to neurological difficulties associated with depression (Figure 1N).

### 2.2 Depression Emerges as a Specific Neurological Complication Resulting from Hematoma in the mPFC Region

**Figure 2:**
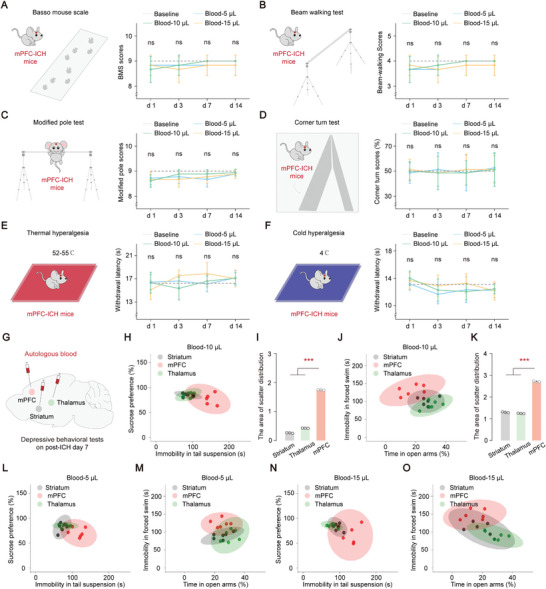
Depression emerges as a specific neurological complication resulting from hematoma in the mPFC region. A–D) Behavioral analysis of hemiplegia on different days with varying volumes of hematoma in the mPFC region. Hemiplegia analysis includes the basso mouse scale (A), beam walking test (B), modified pole test (C), and corner turn test (D). The baseline represents the mean of the control group. Statistical analysis: comparison of different blood groups with the control group on the same day was performed using one‐way ANOVA and Tukey's multiple comparison test. *P* > 0.05 indicates not significant. E,F) Behavioral analysis of sensory dysfunction on different days with varying volumes of hematoma in the mPFC. Sensory dysfunction contains thermal hyperalgesia (E) and cold hyperalgesia (F). The baseline represents the mean of the control group. Statistical analysis: Comparison of different blood groups with the control group on the same day was performed using one‐way ANOVA and Tukey's multiple comparison test. *P* > 0.05 indicates not significant. G) Diagram showing autologous blood injections in three different regions, namely mPFC, striatum, and thalamus, with the same hematoma volume. H–K) Scattered distribution of depression‐related behaviors observed after hematoma at various locations within the Blood 10 µL group (H and J). (I) Area of scatter distribution: “Striatum group” = 0.262, “Thalamus group” = 0.426, “mPFC group” = 1.75. One‐way ANOVA, *P* < 0.0001, Tukey's multiple comparisons test: *P* (Striatum, mPFC) < 0.0001, *P* (Thalamus, mPFC) < 0.0001; (K) Area of scatter distribution: “Striatum group” = 1.301, “Thalamus group” = 1.245, “mPFC group” = 2.711. One‐way ANOVA, *P* < 0.0001, Tukey's multiple comparisons test: *P* (Striatum, mPFC) < 0.0001, *P* (Thalamus, mPFC) < 0.0001. L–O) Scattered distribution of depression‐related behaviors observed after hematoma at various locations within the Blood 5 µL group (L and M) and the Blood 15 µL group (N and O). Data are mean ± sd. In (A‐O), each group, *n* = 6 mice. Not significant (ns) and *P* < 0.001(***).

To assess the severity of hemiplegia and sensory dysfunction associated with various hematoma volumes in mPFC‐ICH, we examined behavioral data and tracked the changes in these behaviors over time. Hemiplegia analysis, which involved using the Basso mouse scale, beam walking test, modified pole test, and corner turn test, did not reveal significant differences between the different hematomas and the control group as the days progressed (*P* > 0.05, **Figure** **2**A–D; Figure S3A–D, Supporting Information). These findings suggest that hematoma in the mPFC region does not lead to hemiplegia as a neurological complication. Similarly, the analysis of sensory dysfunction indicated no significant alterations in thermal hyperalgesia and cold hyperalgesia upon stimulation with diverse hematomas (*P* > 0.05, Figure 2E,F; Figure S4A–D, Supporting Information). This indicates that hematoma in the mPFC has a limited effect on sensory dysfunction. To further explore the characteristics of the three complications, namely hemiplegia, sensory dysfunction, and depression, occurring in response to various hematomas in the mPFC, the change percentages of these complications on specific days were quantitatively evaluated: hemiplegia on day 3, sensory on day 3, and depression on day 7 (Figure S4E, Supporting Information). A substantial change percentage of 50% in depression‐related tests, namely the tail suspension and forced swim tests was observed, while hemiplegia and sensory evaluation manifested less pronounced changes. These findings indicate that depression exhibits a significantly higher susceptibility in the mPFC following hematoma.

In the context of both ICH and the pathophysiology of depression, the frontal lobe, striatum, and thalamus emerge as frequently affected regions.^[^ ^11^ ^]^ To investigate the spatial distribution pattern of depressive indicators in different regions affected by hematoma (mPFC, striatum, and thalamus) (Figure 2G), this study focused on the spatial distribution of depressive‐related behaviors in three hematoma regions (Figure 2H,J,L–O). The data indicates that depression‐related behaviors were more widespread in the mPFC compared to the striatum and thalamus, whereas depression‐related behaviors were more concentrated in the striatum and thalamus (*P* < 0.001, Figure 2I,K; Figure S4F–I, Supporting Information). This result suggests that depression is more likely to occur in the mPFC than in the striatum and thalamus. Thus, these findings strongly show that depression is a specific neurological complication that develops after hematoma in the mPFC, underscoring the importance of considering the impact of mPFC‐ICH on mental health outcomes.

### 2.3 VTA Exhibits a Higher Percentage of Connectivity Disruption Compared to the LHb and STR

**Figure 3:**
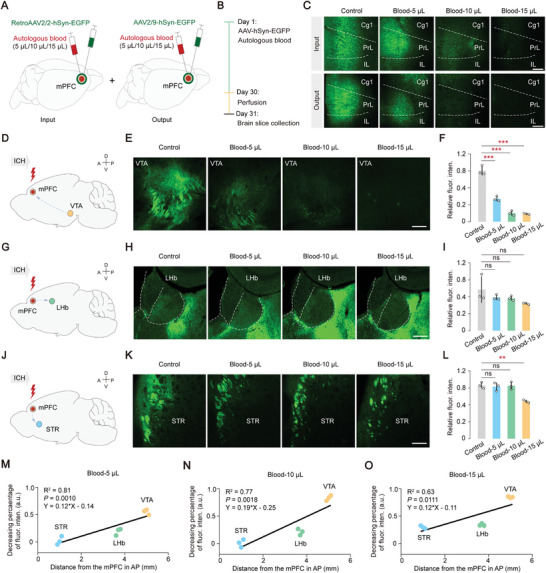
VTA exhibits a higher percentage of connectivity disruption compared to the LHb and STR. A) Schematic diagram illustrating anterograde and retrograde virus injections following hematoma in the mPFC. B) Experimental timeline. C) Expression of anterograde and retrograde virus in injection sites following hematoma in the mPFC. Scale bar, 200 µm. Abbreviation. CG1: Cingulate cortex, area 1; PrL: anterior marginal cortex; IL: marginal hypocortex. D,E) Changes in fluorescence observed in corresponding brain regions along the ventral tegmental area (VTA) – mPFC depression circuit at varying volumes of hematoma. Scale bar, 100 µm. F) Relative fluorescence density (arbitrary units) of VTA near the hematoma in the four different groups. Statistical analysis: one‐way ANOVA, *P* < 0.0001, Tukey's multiple comparisons test: *P* (Control, Blood 5 µL) < 0.0001, *P* (Control, Blood 10 µL) < 0.0001, *P* (Control, Blood 15 µL) < 0.0001. G‐H) Changes in fluorescence observed in corresponding brain regions along the mPFC – lateral habenular nucleus (LHb) depression circuit at varying volumes of hematoma. Scale bar, 100 µm. I) Relative fluorescence density (arbitrary units) of LHb near the hematoma in the four different groups. Statistical analysis: one‐way ANOVA, *P* = 0.1731, Tukey's multiple comparisons test: *P* (Control, Blood 5 µL) = 0.5338, *P* (Control, Blood 10 µL) = 0.4480, *P* (Control, Blood 15 µL) = 0.1328. J,K) Changes in fluorescence observed in corresponding brain regions along the mPFC – striatum (STR) depression circuit at varying volumes of hematoma. Scale bar, 100 µm. L) Relative fluorescence density (arbitrary units) of STR near the hematoma in the different four groups. Statistical analysis: one‐way ANOVA, *P* = 0.0008, Tukey's multiple comparisons test: *P* (Control, Blood 5 µL) = 0.9699, *P* (Control, Blood 10 µL) = 0.9988, *P* (Control, Blood 15 µL) = 0.0015. M–O) Correlation analysis was conducted on the decline percentages of fluorescence density in three brain regions at different distances from the mPFC. A line with linear regression was fitted. (M) Blood 5 µL: R^2^ = 0.81, *P* = 0.0010, Y = 0.12*X – 0.14; (N) Blood 10 µL: R^2^ = 0.77, *P* = 0.0018, Y = 0.19*X – 0.25; (O) Blood 15 µL: R^2^ = 0.63, *P* = 0.0011, Y = 0.12*X + 0.11. In (D‐L), fluorescence density (% area) was analyzed using Default through Image J. Data are mean ± sd. In (D‐O), each group, *n* = 3 mice. Not significant (ns), *P* < 0.01(**), and *P* < 0.001(***).

Next, potential connectivity changes within the mPFC and its network of brain circuits were investigated by using varying injected volumes of hematoma. After inducing hematoma in mice, RetroAAV2/2‐hSyn‐EGFP or AAV2/9‐hSyn‐EGFP viral vectors into their mPFC were injected (**Figure** **3**A,B). Four weeks later, EGFP expression in mPFC neurons near the lesion was observed (Figure 3C). The expression of EGFP decreased as the hematoma volume increased, indicating a significant impact of hematoma volume on the neurons near the lesion (Figure S5A,B, Supporting Information). Previous studies have suggested significant roles played by the STR, LHb, and VTA in information processing related to depression within mPFC circuits.^[^ ^16^, ^17^, ^22^ ^]^ Therefore, assessing their connectivity within the STR, LHb, and VTA were focus. A substantial reduction in fluorescence intensity in the VTA brain regions across various hematoma groups was shown (*P* < 0.001, Figure 3D–F). However, no significant changes in fluorescence intensity were observed in the LHb and STR compared to both the control and blood groups (Figure 3G–L). Interestingly, distinct levels of fluorescence intensity reduction were observed in the three brain areas when exposed to the same hematoma. The VTA exhibited a significant decrease in fluorescence intensity compared to both the LHb and STR (*P* < 0.01), while the decrease in STR was notably lower than that in LHb (*P* < 0.05, Figure S5C, Supporting Information). The decreasing percentage of fluorescence intensity as a function of distance from the mPFC along the anterior‐posterior (AP) axis was explored and an increasing damage percentage with greater distance from the mPFC was revealed (Figure 3M–O). These findings indicate that the VTA exhibits a higher percentage of connectivity disruption compared to the LHb and STR following hematoma in the mPFC, suggesting potential emotional disorders and disturbances in reward function.

### 2.4 Higher Percentages of Loss in Long‐Range Projections Following Hematoma in the mPFC

**Figure 4:**
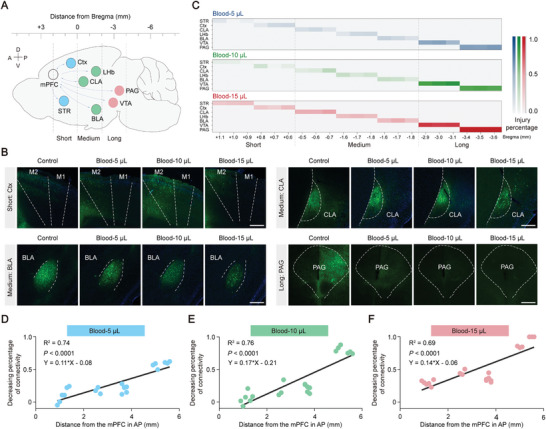
Higher percentages of loss in long‐range projections following hematoma in the mPFC. A) Schematic diagram illustrating the mPFC whole‐brain circuit connection. The distance from the mPFC is classified into three parts: short, medium, and long. Abbreviation. CLA: claustrum; BLA: basal lateral amygdala; THAL: thalamus; PAG: Gray matter around the aqueduct. B) Changes in fluorescence observed in corresponding brain regions in the Ctx, CLA, BLA, and mPFC at varying volumes of hematoma. Scale bar, 100 µm. C) Heat map of the injury percentage for the mPFC whole‐brain circuit connection with varying distances from anterior‐posterior (AP) under different hematoma volumes. D–F) Linear fitted trend of the injury percentage for the mPFC whole‐brain circuit connection at varying distances from AP under different hematoma volumes. A line with linear regression was fitted. (D) Blood 5 µL: R^2^ = 0.74, *P* < 0.0001, Y = 0.11*X – 0.080; (E) Blood 10 µL: R^2^ = 0.76, *P* < 0.0001, Y = 0.17*X – 0.21; (F) Blood 15 µL: R^2^ = 0.69, *P* < 0.0001, Y = 0.14*X – 0.06. In (A–C), each group, *n* = 3 mice.

To determine if this characteristic is consistent throughout all circuits in the mPFC, regions were categorized into three groups based on their distance from the mPFC along the anterior‐posterior axis: short distance (STR: striatum, cortex), medium distance (THAL: thalamus, CLA: claustrum, BLA: basolateral amygdala), and long distance (PAG: periaqueductal gray, VTA: ventral tegmental area) (**Figure** **4**A). A higher injury percentage in the PAG and VTA regions was observed as the distance from the mPFC along the AP axis increased, which is consistent with their longer connectivity. In contrast, a lower injury percentage was noted in the STR and cortex regions, corresponding to their shorter distance from the mPFC (Figure 4B,C). Based on these distinct trends, we hypothesize that there is a positive correlation between the injury percentage of connectivity and the distance from the mPFC. A distinct linear correlation among different hematoma groups in whole‐brain regions has been verified using a comprehensive analysis (Figure 4D,F; Blood‐5 µL: R^2^ = 0.74, *P* < 0.0001; Blood‐10 µL: R^2^ = 0.76, *P* < 0.0001; Blood‐15 µL: R^2^ = 0.69, *P* < 0.0001). In summary, our findings reveal an unforeseen role of mPFC distance in the mPFC circuits, with long‐range projections exhibiting a higher percentage of loss following hematoma in the mPFC, indicating that extended damage beyond the site of hematoma may contribute to the extensive impairments in cognition, emotion, and behavior.

### 2.5 mPFC‐Hematoma Neurons Exhibit a Shorter Duration and Faster Decay of Action Potentials

**Figure 5:**
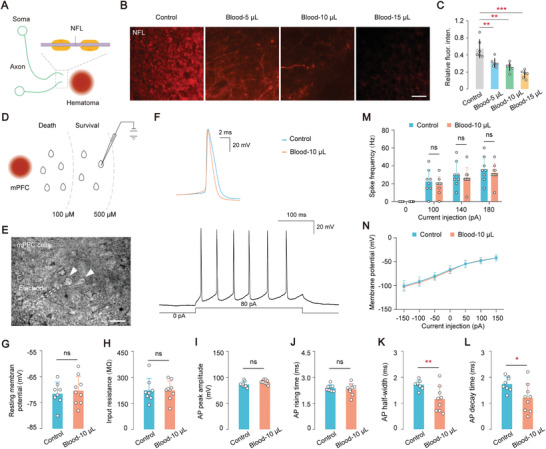
mPFC‐hematoma neurons exhibit a shorter duration and faster decay of action potentials. A) Diagram depicting the location of neurofilament light chain (NFL) within axons. B) Immunohistochemistry images showing the morphological changes of NFL near the hematoma in different groups. Scale bar, 20 µm. C) Relative fluorescence density (arbitrary units) of NFL near the hematoma in the four different groups. Statistical analysis: one‐way ANOVA, *P* < 0.0001, Tukey's multiple comparisons test: *P* (Control, Blood 5 µL) = 0.0032, *P* (Control, Blood 10 µL) = 0.0002, *P* (Control, Blood 15 µL) < 0.0001. Fluorescence density (% area) was analyzed using Default through Image J. D) A scheme depicting the whole‐cell patch‐clamp recording in vitro. E) Acute slice used for whole‐cell patch‐clamp recording in vitro. Scale bar: 30 µm. Responses of these cells to injected current pulses (−150, −100, −50, 0, 50, 100, 150 pA) were recorded. F) Typical traces of spontaneous action potentials (sAPs) in mPFC neurons from control and blood groups on post‐ICH day 3. G) Comparison of the resting membrane potential showed no significant difference between the control group (−71.5 mV) and the Blood‐10 µL group (−70.3 mV) (*P* = 0.64, unpaired t‐tests). H) Comparison of the input resistance revealed no significant difference between the control group (225.5 MΩ) and the Blood‐10 µL group (225.33 MΩ) (*P* = 0.99, unpaired t‐tests). I) Comparison of the AP peak amplitude showed no significant difference between the control group (86.65 mV) and the Blood‐10 µL group (91.29 mV) (*P* = 0.06, unpaired t‐tests). G) Comparison of the AP rising time showed no significant difference between the control group (2.39 ms) and the Blood‐10 µL group (2.32 ms) (*P* = 0.57, unpaired t‐tests). K) Comparison of the AP half‐width. “Control group”: 1.71, “Blood‐10 µL group”: 1.17. *P* (Control, Blood‐10 µL) = 0.0085, unpaired t‐tests (parametric tests). L) Comparison of the AP decay time. “Control group”: 1.74, “Blood‐10 µL group”: 1.21. *P* (Control, Blood‐10 µL) = 0.024, unpaired t‐tests (parametric tests). M) Comparison of the spike frequency. 100 pA: “Control group”: 22.5, “Blood‐10 µL group”: 18.33; 140 pA: “Control group”: 30, “Blood‐10 µL group”: 26.67; 180 pA: “Control group”: 36.25, “Blood‐10 µL group”: 32.22. 100 pA: *P* (Control, Blood‐10 µL) = 0.4746, unpaired t‐tests (parametric tests); 140 pA: *P* (Control, Blood‐10 µL) = 0.7102, unpaired t‐tests (nonparametric tests); 180 pA: *P* (Control, Blood‐10 µL) = 0.5128, unpaired t‐tests (parametric tests). N) I–V curves of two group data. Data are mean ± sd. In (A–C), each group, n = 6 mice. In (D‐N), the control group consisted of eight neurons recorded from three mice, blood group consisted of nine neurons recorded from three mice. Not significant (ns), *P* < 0.05(*), *P* < 0.01(**), and *P* < 0.001(***).

To observe subcellular‐level changes near the hematoma, neurofilaments as the primary components of axons (**Figure** **5**A),^[^ ^23^ ^]^ a reduction in fluorescence intensity of the neurofilament light chain (NFL) was observed near the lesion in different hematoma groups, with more pronounced damage occurring in larger hematoma groups (Figure 5B). A positive correlation was found between hematoma volume and NFl levels in the vicinity of the lesion area (Figure 5C). To investigate the electrophysiological characteristics of mPFC neurons near the hematoma, in vitro patch‐clamp recordings on both mPFC and mPFC‐hematoma neurons were conducted (Figure 5D,E). Brain slices were obtained on day 2, day 3, and day 4 after the induction of hematoma, and recordings were performed on neurons located within a range of 100–500 µM from the hematoma (Figure 5F). Neurons located within a very close range (within 100 µM) of the hematoma were largely nonviable (cell death) and were excluded from the analysis (Figure 5D). Comparing the electrophysiological properties of the control group with the blood 10 µL group at a significance level of α = 0.05, no significant differences were observed in resting membrane potential (Vm), input resistance (Rin), AP peak amplitude, and AP rising time (Figure 5G–J). However, a decrease in both AP half‐width (*P* = 0.0085) and AP decay time (*P* = 0.024) was observed in the blood 10 µL group compared to the control group (Figure 5K,L), indicating that the presence of hematoma leads to shorter duration and faster decay of action potentials in mPFC‐hematoma neurons. Furthermore, the AP frequency and the current‐voltage characteristic (I‐V) curve showed no significant differences (Figure 5M,N). These findings suggest that mPFC‐hematoma neurons exhibit distinct electrophysiological characteristics compared to normal neurons, specifically in terms of the AP half‐width and AP decay time, resulting in decreased neuronal excitability and alterations in synaptic communication that further impact neural circuits associated with emotion regulation and cognitive function.

### 2.6 Targeting the JAK‐STAT Pathway as a Potential Intervention for Depressive Symptoms Resulting from Hematoma in the mPFC

**Figure 6:**
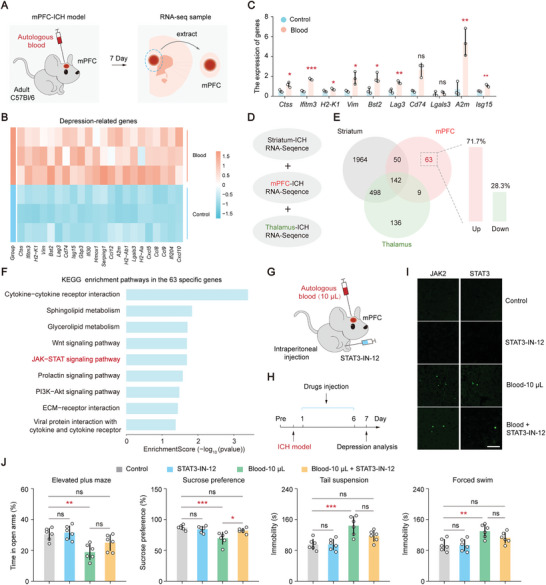
Targeting the JAK‐STAT pathway as a potential intervention for depressive symptoms resulting from hematoma in the mPFC. A) A diagram of RNA transcriptional sequencing analysis of hematoma in the mPFC on day 7. B) Heatmaps of depression‐related DEG expressed in control and blood groups. C) RT‐PCR analysis of depression‐related genes in control and blood groups showed the following results: *P* (*Ctss*) = 0.0160, *P* (*Ifitm3*) < 0.001, *P* (*H2‐K1*) = 0.0486, *P* (*Vim*) = 0.0225, *P* (*Bst2*) = 0.0160, *P* (*Lag3*) = 0.0035, *P* (*Lgals3*) = 0.4993, *P* (*A2m*) = 0.0059, *P* (*Isg12*) = 0.0023 (unpaired t‐tests, parametric tests); *P* (*Cd74*) = 0.1000 (unpaired t‐tests, nonparametric tests). D) Schematic diagram illustrating the screening of specific genes using three different ICH transcriptome datasets. E) Venn diagram comparing the differential expression of genes. The right panel shows the proportion of upregulated and downregulated genes among the 63 specific genes in the mPFC. F) KEGG enrichment pathways in the 63 specific genes after hematoma in the mPFC. G) Behavioral evaluation diagram of drug injection. H) Experimental timeline. I) Immunofluorescence images showing the expression of JAK2 and STAT3 proteins in various groups after the injection of JAK‐STAT pathway inhibitors. Bar, 100 µm. J) Behavioral analysis of depression conducted on day 7 showed the following results (one‐way ANOVA and Tukey's multiple comparison test): For elevated plus maze, *P* = 0.0006, *P* (Control, STAT3‐IN‐12) = 0.9806, *P* (Control, Blood 10 µL) = 0.0024, *P* (Control, Blood + STAT3) = 0.2256, *P* (Blood 10 µL, Blood + STAT3) = 0.1606; for sucrose preference, *P* = 0.0008, *P* (Control, STAT3‐IN‐12) = 0.8873, *P* (Control, Blood 10 µL) = 0.0009, *P* (Control, Blood + STAT3) = 0.5683, *P* (Blood 10 µL, Blood + STAT3) = 0.0166; for tail suspension, *P* = 0.0003, *P* (Control, STAT3‐IN‐12) > 0.9999, *P* (Control, Blood 10 µL) = 0.0006, *P* (Control, Blood + STAT3) = 0.1734, *P* (Blood 10 µL, Blood + STAT3) = 0.1737; for forced swim, *P* = 0.0017, *P* (Control, STAT3‐IN‐12) > 0.9999, *P* (Control, Blood 10 µL) = 0.0039, *P* (Control, Blood + STAT3) = 0.1991, *P* (Blood 10 µL, Blood + STAT3) = 0.2599. Data are mean ± sd. In (C) and (I), each group, *n* = 3 mice. In (J), each group, *n* = 6 mice. Not significant (ns), *P* < 0.05(*), *P* < 0.01(**), and *P* < 0.001(***).

Neurological complications, particularly depression induced by hematomas, are a primary concern in mPFC‐ICH mice. Therefore, our objective was to characterize the transcriptional expression profile of the mPFC in mice on post‐ICH day 7, using RNA sequencing as the primary method (**Figure** **6**A). Statistical analysis revealed that out of 266 differentially expressed genes, 236 (88.7%) were significantly upregulated, while 30 genes were downregulated (Figure S6A, Table S2, Supporting Information). Gene Ontology (GO) and Kyoto Encyclopedia of Genes and Genomes (KEGG) enrichment analysis of the RNA‐seq data demonstrated significant enrichment of depression‐related pathways on day 7. These pathways included the response to interferon−gamma, antigen processing and presentation, and regulation of immune effector processes (Figure S6B–E, Supporting Information). Notably, a substantial number of upregulated genes associated with depression were identified, such as *Ctss*, *Ifitm3*, *H2‐K1*, *Vim*, *Bst2*, *Lag3*, *Cd74*, *Isg15*, *Gbp3*, and *Cxcl10* (Figure 6B; Table S3, Supporting Information). The transcriptional expression patterns of these key genes in hematoma were validated using Quantitative Real‐time PCR (Figure 6C; Table S7, Supporting Information). These findings suggest that these genes may play a critical role in the onset and progression of depression induced by ICH.

Our next objective was to investigate specific signaling pathways contributing to the neurological complications of depression following hematoma in the mPFC. By comparing the differential transcription expression profiles among three ICH areas (striatum, mPFC, and thalamus, Figure 6D), 63 differentially expressed genes unique to the mPFC groups were identified (Table S4, Supporting Information), with the majority (71.7%) of these genes being upregulated (Figure 6E). To examine the enrichment pathway of these 63 specific genes, Gene Ontology (GO) enrichment analysis was performed using a significance level of α = 0.05, which includes the biological process (BP, Figure S7A, Table S5, Supporting Information), cellular component (CC, Figure S7B, Table S5, Supporting Information), and molecular function (MF, Figure S6C, Table S5, Supporting Information). The biological process analysis revealed a significant enrichment (*P* < 0.05) of the JAK‐STAT signaling pathway. Consistently, KEGG enrichment analysis also demonstrated a significant enrichment (*P* < 0.05) of the JAK‐STAT signaling pathway in the mPFC‐specific genes (Figure 6F; Table S6, Supporting Information). These findings collectively suggest that the JAK‐STAT signaling pathway may be involved in mediating the effects of hematoma in the mPFC and could be a potential target for intervention in depressive symptoms resulting from hematoma. Furthermore, significant enrichment was observed in several pathways, including the non‐canonical Wnt signaling pathway and positive regulation of the non‐canonical Wnt signaling pathway (Figure S7A, Supporting Information). Additionally, pathways related to interferon‐beta, such as response to interferon‐beta and cellular response to interferon‐beta (Figure S7A, Supporting Information), as well as pathways associated with interferon‐gamma, such as response to interferon‐gamma and cellular response to interferon‐gamma (Figure S6B, Supporting Information), also exhibited notable enrichment. These findings strongly suggest a potential correlation between the aforementioned pathways and the development of depression following mPFC hematoma.

The precise function of the JAK‐STAT signaling pathway in depression after hematoma in the mPFC is still not fully understood. To investigate its involvement in the development of depression‐related neurological complications, the JAK‐STAT signaling pathway inhibitor (STAT3‐IN‐12) was administered via intraperitoneal injection in ICH mice and depression‐related behavioral tests were conducted on day 7 (Figure 6G,H). Immunofluorescence tests showed significantly higher fluorescence intensity of JAK2 and STAT3 in the blood 10 µL group on day 7 compared to the control and STAT3‐IN‐12 groups (*P* < 0.001), suggesting the activation of the JAK‐STAT signaling pathway after hematoma in the mPFC (Figure 6I; Figure S7D, Supporting Information). Furthermore, the expression of JAK2 and STAT3 in the STAT3‐IN‐12 group was similar to that of the control group, indicating that the inhibitor did not significantly impact the JAK‐STAT signaling pathway (*P* > 0.05). However, STAT3‐IN‐12 significantly reduced the fluorescence intensity of JAK2 and STAT3 in the blood 10 µL group (*P* < 0.001), suggesting its inhibitory effect on the expression of JAK2 and STAT3 compared to the blood 10 µL group. To investigate the contribution of the JAK‐STAT signaling pathway to depression, depression‐related behavior tests were conducted in various experimental groups (Figure 6J). These results indicated that STAT3‐IN‐12 had little impact on the relevant behaviors (*P* > 0.05). However, mice treated with 10 µL of blood exhibited significant alterations in behavior during the elevated plus maze, sucrose preference, tail suspension, and forced swim tests (*P* < 0.01). Compared to the group receiving 10 µL of blood, the inhibitor significantly changed sucrose preference in ICH mice (*P* < 0.001). Additionally, applying STAT3‐IN‐12 in ICH mice brought depression‐related behaviors closer to those observed in the control group (*P* > 0.05), suggesting that targeting the JAK‐STAT pathway may alleviate the severity of depression in mPFC‐ICH. These findings open up avenues for future research aimed at developing novel therapeutic strategies for mitigating the psychiatric consequences of frontal lobe hemorrhage.

## 3 Discussion

After a stroke, the prevalence of depressive symptoms ranges from approximately 30% to 50%, causing various negative impacts on patients, especially in cases of frontal lobe hemorrhage.^[^ ^13^ ^]^ However, the underlying mechanisms responsible for depression following frontal lobe hemorrhage remain poorly understood, highlighting the need to investigate this knowledge gap. In this study, we conducted a series of comprehensive experiments, and our findings are as follows: (1) Depression is a specific neurological complication following mPFC hematoma. (2) VTA exhibits a higher percentage of connectivity damage compared to LHb and STR after hematoma, with a higher loss percentage observed in long‐distance projections from mPFC. (3) mPFC neurons affected by hematoma demonstrate shortened action potential duration and accelerated decay. (4) Transcriptomic analysis revealed a JAK‐STAT signaling pathway associated with mPFC‐ICH, and targeting this pathway mitigated the severity of post‐ICH depression. These findings shed light on the pathogenesis of depression following mPFC hematoma and provide a potential therapeutic target for alleviating post‐ICH depression.

The volume of hematoma serves as a significant indicator for assessing poor prognosis in patients with ICH.^[^ ^24^ ^]^ A study revealed a significant positive correlation between hematoma volume and depression scores in post‐hemorrhagic patients,^[^ ^25^ ^]^ suggesting that larger hematomas are associated with increased severity of depressive symptoms. Consistently, our animal experimentation demonstrated a positive correlation between hematoma volume and the severity of depressive symptoms. This association may be attributed to the physiological and biochemical alterations resulting from increased hematoma volume, including inflammatory responses, neuronal death, and cerebral tissue damage,^[^ ^26^ ^]^ which contribute to more severe neurological impairment and diminished quality of life. Furthermore, hematoma volume significantly influences the temporal progression of depressive symptoms, as evidenced by distinct differences in depression‐related behaviors observed among mice in different hematoma groups during the early stages (Day 7 and Day 14). These findings emphasize the need to consider the impact of hematoma volume when investigating and treating post‐frontal lobe hemorrhage‐related depressive symptoms.

Several circuits in the frontal lobe have been reported to be associated with depression, namely the VTA‐mPFC, mPFC‐LHb, and mPFC‐STR circuits.^[^ ^15^, ^16^, ^17^ ^]^ In the three circuits associated with depression, our study revealed that the connectivity disruption was most severe in the VTA‐mPFC circuit. The VTA‐mPFC circuit has been linked to emotional regulation and reward systems, with the VTA serving as a primary source of dopamine neurons and the mPFC involved in emotional regulation, decision‐making, and reward processes.^[^ ^27^ ^]^ Therefore, the disruption of connectivity between these two regions suggests the potential for emotional disorders and disturbances in reward function. Previous research has indicated that functional abnormalities in the VTA‐mPFC circuit are associated with psychiatric disorders such as depression, schizophrenia, and addictive behaviors.^[^ ^28^ ^]^ The significant disruption of the VTA‐mPFC connection observed in frontal lobe hematoma has important implications for the clinical management and treatment of patients, as it contributes to the development of depressive mood disorders and guides effective interventions. In addition, previous studies have demonstrated that the locus coeruleus (LC) and raphe nuclei are critical serotonergic and noradrenergic nuclei in the brainstem, implicated in emotion, cognition, and affective regulation.^[^ ^29^ ^]^ Particularly, their close projections to the mPFC suggest that the interaction between LC/raphe nuclei and the mPFC may play a significant role in the development and modulation of depressive and anxiety symptoms.^[^ ^30^ ^]^ Future investigations should further explore the projection patterns between LC/raphe nuclei and the mPFC following ICH, as well as their involvement in the manifestation of depressive and anxiety disorders. Such endeavors will provide a more comprehensive understanding of the neurobiological mechanisms underlying post‐stroke depression and anxiety.

Analysis of the brain's overall connectivity also revealed a pattern of circuit damage caused by frontal lobe hematoma in the mouse model, characterized by a higher loss percentage in long‐distance projections. Similar effects of long‐distance connectivity damage have been reported in various neurological diseases. For instance, studies on epilepsy have shown that long‐distance connectivity damage is associated with seizure frequency and severity.^[^ ^31^ ^]^ In Alzheimer's disease, the disruption of long‐distance connections in the frontal lobe is related to cognitive decline and disease progression.^[^ ^32^ ^]^ In our study, we found that the VTA and PAG exhibited higher percentages of long‐distance connection damage in the frontal lobe. The VTA‐mPFC circuit is closely related to emotional regulation and reward systems,^[^ ^15^ ^]^ while the mPFC‐PAG circuit plays a crucial role in emotional regulation and pain control.^[^ ^33^ ^]^ Therefore, the observed damage to the VTA‐mPFC circuit, involved in emotional regulation and reward systems, and the mPFC‐PAG circuit, crucial for emotional regulation and pain control, suggests that frontal lobe hematoma is likely to impair emotional regulation and pain control functions, potentially contributing to the development of emotional disorders and pain symptoms. When assessing and managing patients with frontal lobe hemorrhage in clinical practice, healthcare professionals should consider the possibility of long‐distance damage and take appropriate intervention measures to promote optimal recovery and functional rehabilitation for patients.

In this study, we investigated the AP characteristics of surviving neurons within a range of 100–500 µm from the frontal lobe. The results revealed significant electrophysiological alterations in the mPFC following hematoma, characterized by a significant reduction in AP duration and an increased rate of AP decay. The observed shortened duration of APs may episodes of reflect transient abnormal activity in the mPFC,^[^ ^34^ ^]^ while the accelerated decay indicates disturbances in network dynamics within the mPFC,^[^ ^35^ ^]^ collectively compromising neuronal function and potentially contributing to impaired emotional regulation. Previous research has consistently implicated factors such as neuronal damage, inflammation, synaptic alterations, ion channel modifications, and disruptions in neurotransmitter systems associated with shortened AP duration and accelerated decay,^[^ ^35^, ^36^ ^]^ thereby highlighting their potential relevance to the electrophysiological changes observed in frontal lobe hematoma.

Furthermore, we identified specific JAK‐STAT signaling pathways in the mPFC through transcriptomic analysis. Targeting the JAK‐STAT pathway in animal models was found to reduce the severity of depression in cases of ICH. The JAK‐STAT signaling pathway is an important cellular signaling pathway involved in regulating processes such as cell proliferation, differentiation, and immune response.^[^ ^37^ ^]^ Its relationship with JAK‐STAT signaling has been reported in various immune system disorders (e.g., rheumatoid arthritis, systemic lupus erythematosus), tumors (e.g., leukemia, lymphoma), and hematological disorders (e.g., myelofibrosis).^[^ ^38^ ^]^ A study investigating the molecular mechanisms of bipolar disorder (BPD) employed RNA‐seq data from the prefrontal cortex (PFC), including post‐mortem samples derived from individuals with BPD and matched control subjects.^[^ ^39^ ^]^ The results revealed significant alterations in the interplay between the JAK‐STAT pathway and the mTOR pathway within the PFC of individuals with bipolar disorder. Although the primary focus of this study centered on bipolar disorder, the evidence derived from post‐mortem samples suggests the potential presence of changes within the JAK‐STAT pathway in the brains of individuals with depression. Furthermore, an additional bioinformatic analysis, based on drug targets, also identified pathways implicated in depression, including the JAK‐STAT pathway.^[^ ^40^ ^]^ Our study further demonstrates the involvement of the JAK‐STAT signaling pathway in depression induced by frontal lobe hematoma. However, in post‐stroke depression, the downstream targets and regulatory effects of the JAK‐STAT pathway warrant further investigation. Currently, studies have reported common downstream targets of the JAK‐STAT pathway, including SOCS3, PIAS, PTPs, and Bcl‐2, which play important roles in cellular proliferation, differentiation, survival, and inflammation.^[^ ^41^ ^]^ Transcriptomic data indicates differential expression of *SOCS3* and *Ptpn6* among these downstream targets (Figure S7E, Supporting Information). Moreover, the downstream target SOCS3 has been implicated in depression.^[^ ^42^ ^]^ Therefore, we speculate that the JAK‐STAT pathway may exert its regulatory effects on post‐stroke depression through the modulation of SOCS3 or Ptpn6. Subsequent studies could selectively manipulate the expression or activity of specific downstream targets to enhance our understanding of the regulatory mechanisms of the JAK‐STAT pathway. In addition, studies have shown that neurochemical signaling, synaptic function, and neuronal activity change at the onset of depression.^[^ ^43^ ^]^ However, the specific effects of the JAK‐STAT signaling pathway on electrophysiology have not been extensively investigated. Subsequent investigations could explore the impact of JAK‐STAT pathway activation on neuronal firing, synaptic transmission, and other electrophysiological aspects, thereby elucidating the mechanisms through which the JAK‐STAT signaling pathway regulates depression through electrophysiological modulation.

There are two limitations to this study. The first limitation is that, although our study focused on the JAK‐STAT signaling pathway, we acknowledge the importance of considering other confounding variables and alternative explanations. First, transcriptomic data indicates a certain level of enrichment for pathways such as interferon‐gamma and interferon‐beta, which are associated with depression.^[^ ^44^ ^]^ Therefore, investigating the interactions between the JAK‐STAT pathway and other signaling pathways related to depression can be explored. This could be achieved through targeted pharmacological interventions or genetic manipulations in animal models, allowing us to elucidate the synergistic or antagonistic effects between these pathways. Second, exploring potential compensatory mechanisms that may counteract or modulate the depressive effects of frontal lobe hematoma is also crucial. Longitudinal studies assessing changes in circuit connectivity, cellular activity, and gene expression over some time following hematoma would provide insights into the dynamic nature of these compensatory mechanisms. Additionally, it is important to consider factors such as genetic variability and individual differences in vulnerability to depression. Conducting studies with larger sample sizes and stratifying participants based on genetic or clinical characteristics could help identify subgroups with distinct responses to frontal lobe hemorrhage and depression. The second limitation is related to the animal model of hemorrhagic stroke. The autologous blood model serves as a common experimental model for studying intracerebral hemorrhage.^[^ ^45^ ^]^ The results obtained from this model align with clinical observations, such as the differential expression of the JAK‐STAT pathway in post‐mortem samples, providing a theoretical basis for clinical applications to some extent. However, the clinical manifestation of hemorrhagic stroke requires consideration of additional factors. First, in our autologous blood model, the hematoma formation is controlled and localized within the mPFC, whereas hemorrhagic stroke can affect multiple regions beyond the mPFC. The size, location, and specific vascular damage may differ between the two conditions. Second, our study primarily focused on the local effects within the mPFC and its associated circuitry in the hematoma model, while hemorrhagic stroke can induce systemic changes such as fluctuations in blood pressure and interruption of cerebral blood flow.^[^ ^46^ ^]^ Third, it should be noted that the gene expression profile observed in the hematoma model may not fully reflect the complex and dynamic changes observed in clinical hemorrhagic stroke. The extent and nature of gene transcription alterations may differ between the two conditions. Lastly, although we investigated the involvement of the JAK‐STAT signaling pathway and its modulation as a potential therapeutic strategy in our study, the effectiveness and applicability of such interventions may differ between the hematoma model and clinical hemorrhagic stroke. The efficacy and suitability of intervention measures need to consider additional factors, including systemic influences and individual characteristics.

## 4 Conclusion

This study provides valuable insights into the specific patterns of disrupted brain connectivity, electrophysiological alterations, and the role of the JAK‐STAT signaling pathway in depression induced by frontal lobe hematoma, shedding light on potential mechanisms underlying the pathology. These findings offer promising therapeutic targets, including the modulation of JAK‐STAT signaling and the restoration of brain connectivity, which hold the potential for developing more effective interventions and treatment approaches tailored specifically for patients with depression following frontal lobe hemorrhage. These targeted interventions may help alleviate depressive symptoms and improve overall patient outcomes.

## 5 Experimental Section

Experimental details are provided in the Supporting Information

## Conflict of Interest

The authors declare no conflict of interest.

## Author Contributions

S.H. and Y.W. designed the experiments; Y.W. and J.D. performed the experiments; Y.W., J.D., and J.M. performed the data analysis; S.H., Y.C., and B.W. inspected the data and evaluated the findings; S.H. and B.W. wrote the manuscript with the help from all authors.

## Supporting information

Supporting Information

Supplementary Table S1

Supplementary Table S2

Supplementary Table S3

Supplementary Table S4

Supplementary Table S5

Supplementary Table S6

Supplementary Table S7

## Data Availability

The data that support the findings of this study are available from the corresponding author upon reasonable request.
