# Supplementary material for: Unraveling the Pathogenesis of Post‐Stroke Depression in a Hemorrhagic Mouse Model through Frontal Lobe Circuitry and JAK‐STAT Signaling
Source: Adv Sci (Weinh). 2024 Jul 1;11(33):2402152. doi: 10.1002/advs.202402152 (PMC11434213; doi:10.1002/advs.202402152)
Supplement: Supplementary file 1 — Supporting Information [file ADVS-11-2402152-s001.pdf]

ADVANCED SCIENCE
Open Access

Supporting Information

for *Adv. Sci.*, DOI 10.1002/advs.202402152

Unraveling the Pathogenesis of Post-Stroke Depression in a Hemorrhagic Mouse Model through Frontal Lobe Circuitry and JAK-STAT Signaling

Yingqing Wu, Jia Deng, Jinsong Ma, Yujie Chen, Ning Hu, Shilei Hao and Bochu Wang**

Unraveling the Pathogenesis of Post-Stroke Depression in a Hemorrhagic Mouse Model through Frontal Lobe Circuitry and JAK-STAT Signaling

Yingqing Wu, Jia Deng, Jinsong Ma, Yujie Chen, Ning Hu, Shilei Hao [*], Bochu Wang [*]

Yingqing Wu, Jinsong Ma, Ning Hu, Shilei Hao, Bochu Wang

Key Laboratory of Biorheological Science and Technology, Ministry of Education, College of Bioengineering, Chongqing University, Chongqing 400030, China

E-mail: shilei_hao@cqu.edu.cn (S. Hao), wangbc2000@126.com (B. Wang)

Jia Deng

College of Environment and Resources, Chongqing Technology and Business University, Chongqing 400030, China

Yujie Chen

Department of Neurosurgery and State Key Laboratory of Trauma, Burn and Combined Injury, Southwest Hospital, Third Military Medical University (Army Medical University), Chongqing, 400038, China

Supplementary Text

5. Experimental Section

5.1 Animals

Adult male C57BL/6J mice (8 weeks, weighing 22-24g) were used in this study, and all experimental procedures were approved by the Institutional Animal Care and Use Committee of Chongqing University. The guidelines prescribed by the NIH Guide for the Care and Use

of Laboratory Animals (National Academies Press, 2011) were followed. The mice were group-housed in a room with a 12-hour light/dark cycle and had ad libitum access to food and water. Random assignment to different groups was conducted using simple randomization based on chance. Surgeries and outcome assessments were conducted by blinded investigators. The rationale for employing young male mice in our study is as follows: 1. Existing research on post-stroke depression or neurological complications often utilizes adult male mice to study underlying mechanisms and therapeutic interventions [1]. By employing similar experimental animals and methodologies, we can compare our findings with the existing literature, facilitating the interpretation and translatability of our research results. 2. Eight-week-old mice were chosen for this study, partly due to their developmental stage being consistent with that of mature mice. During this stage, neural circuits and synaptic connections are stable, allowing us to investigate the effects of unilateral mPFC hematoma on circuit connectivity and electrophysiological properties. 3. Female mice were selected primarily because they commonly exhibit hormonal fluctuations due to estrous cycles. By using male mice, we can minimize the confounding effects of these hormonal fluctuations, enabling us to focus on the direct impact of unilateral mPFC hematoma.

5.2 Stereotaxic administration of adeno-associated viral vectors

To ensure precise injection of the virus into specific target sites and eliminate the impact of various injection sites, a stereotaxic method was employed. The mice were anesthetized with 1%-2% isoflurane in oxygen and secured in a stereotactic head frame with a heating pad maintained at 37℃-38℃. A small craniotomy of 0.5×0.5 mm was performed using a dental drill. Analysis was conducted on selected specific brain regions, namely the mPFC, striatum, and thalamus. On one hand, these regions are commonly affected by cases of ICH [2]. On the other hand, they are associated with the pathophysiology of depression. The mPFC is involved in emotion regulation, cognitive processes, and reward-related behaviors [3]. The

striatum plays a crucial role in reward processing and motivation [4]. The thalamus is involved in regulating arousal, sleep, and emotions [4a, 5]. All mice received injections into different brain areas using the following stereotaxic coordinates: Striatum (AP: +0.80 mm; ML: -2.10 mm; DV: -3.00 mm), mPFC (AP: +1.78 mm; ML: -0.30 mm; DV: -1.85 mm), thalamus (AP: -1.94 mm; ML: +1.50 mm; DV: -3.00 mm) [6]. For virus tracing experiments, 150 nL of the virus was slowly injected into the right brain areas. The injection electrode was slowly inserted into the corresponding brain region at a descent speed of 0.01 mm/s. The virus injection rate was 50 nL/min, followed by a 10~15 minute pause to allow sufficient absorption of the virus. Subsequently, the injection electrode was retracted at a speed of 0.01 mm/s. Proper postoperative care was provided until the animals fully recovered. RetroAAV2/2-hSyn-EGFP (Shanghai Taitool Bioscience Co., Ltd) was used for upstream tracing experiments, whereas AAV2/9-hSyn-EGFP (Shanghai Taitool Bioscience Co., Ltd) was used for downstream tracing experiments. The experiments were conducted within a time frame of approximately 3-5 weeks post-injection.

5.3 Autologous blood-induced ICH model in mice

To minimize variability in hematoma sites, we utilized a stereotaxic method to inject autologous blood at a consistent location in all ICH mice. Autologous blood was obtained from the mice’s tail artery during the procedure [6]. The administered blood volumes were 5 μL, 10 μL, and 15 μL. To ensure precise injection volumes, the injection electrode was labeled with scales corresponding to 5 μL, 10 μL, and 15 μL. The respective blood volumes were drawn and slowly administered into the targeted areas using the same stereotaxic coordinates as the virus injection procedures. The control group was the sham group, which received needle insertion without any actual injection. The vertical and right-sided unilateral injection method was employed for all the aforementioned cases. The injection electrode was slowly inserted into the corresponding brain region at a descent speed of 0.01 mm/s. The

autologous blood injection was performed at a rate of 5 μL/min [7], followed by a 10-minute pause. Subsequently, the injection electrode was retracted at a speed of 0.01 mm/s. Post-surgery, appropriate care was provided, including placing the mice in a heated cage at 36 ~ 37 ℃ for 1-4 hours [6]. No adverse events were observed during the study. Animal monitoring was conducted twice daily, and health was assessed through weight measurements (twice weekly) as well as monitoring food and water intake. We selected the right mPFC as the lesion site for the following reasons: 1. Focusing on a unilateral lesion allowed us to investigate the direct impact of the lesion on the right mPFC without confounding effects from bilateral lesions or interactions between the two hemispheres. 2. Previous studies have consistently highlighted the significant role of the right mPFC in emotional regulation and the development of depressive symptoms [8]. Therefore, by targeting the right mPFC, our research aimed to specifically examine the effects of the lesion on this region and elucidate the underlying mechanisms associated with post-hemorrhagic depression.

5.4 Experimental timelines in virus tracing experiments

Using the stereotaxic method, we injected different volumes of autologous blood (5 μL, 10 μL, and 15 μL) into the brain region of mice, followed by an injection of 150 nL of the anterograde or retrograde virus at the same location. In the control group, we injected the same volume (150 nL) of anterograde or retrograde virus at the same location without any autologous blood injection. The virus tracing experiments consisted of eight groups (upstream: control, blood-5 μL, blood-10 μL, blood-15 μL; downstream: control, blood-5 μL, blood-10 μL, blood-15 μL), each containing 6-8 mice. Since it takes approximately 30 days for the virus to fully express, the mice were perfused after this period. One portion of the mice was utilized for subsequent morphological virus analysis, including whole-brain projection (each group contains 3-4 mice), while the other part of the mice was reserved for later histochemical staining analysis (each group also contains 3-4 mice).

5.5 Histology analysis

For all anatomy experiments, the animals were euthanized following surgery, and perfusion was performed through the left ventricle using a solution of 0.9% saline and 4% paraformaldehyde (PFA). Subsequently, the brains were removed and immersed in 4% PFA overnight. Brain samples were obtained utilizing a cryostat, which produced 40 μm thick slices. DAPI staining was applied to visualize the nucleus. The slices were carefully placed onto glass slides, and a high-powered microscope equipped with a 10× water objective was used to capture images. To map the topography of input or output connectivity, anterior-posterior positions in millimeters from each brain area (all relative to Bregma) were selected. The best matches with the histology atlas were chosen for analysis. Image J was utilized to identify the location of the labeled cells expressing enhanced green fluorescent protein (EGFP). The anterior-posterior (AP) coordinates used for histological analysis of each brain region are as follows: Striatum (+1.34 mm), M1/M2 (+0.26 mm), CLA (-0.58 mm), BLA (-1.46 mm), LHb (-1.82 mm), VTA (-2.92 mm), and PAG (-3.52 mm). For each animal, a series of consecutive sections were collected, including 5-10 sections before and after the AP coordinate corresponding to each brain region. Subsequently, the brain section closest to the targeted brain region's AP coordinate was selected for further analysis. In addition, it has been reported that the circuit connections between VTA, LHb, STR, and mPFC play a significant role in regulating emotions and motivation [9]. VTA serves as a major source of dopamine neurons, which play an important role in emotion regulation [10]. LHb is considered a key structure in negative regulation, and its excessive activity is associated with the pathogenesis of depression [11]. The striatum is a core component of the reward system and is associated with abnormalities in reward function [12]. Therefore, the focus of this study is to analyze the abnormalities in these circuits to explore the pathogenesis of post-stroke depression. By

integrating data from histological slices, the average fluorescence intensity was calculated and normalized across all regions, resulting in a normalized relative fluorescence intensity.

5.6 Cresyl Violet (CV) staining

Viral injection and autologous blood injections of various volumes were performed in the frontal lobe region, followed by perfusion after 24 hours. Frozen brain sections, 4-6 slices (40 μm), were collected before and after the injection site for CV staining. The staining dye used was cresyl violet (MACKLIN, C861450). The staining procedure was performed as follows: sections were deparaffinized in xylene twice for 3 minutes each, followed by dehydration in descending concentrations of ethanol for 3 minutes each step. The sections were rinsed in distilled water until clear droplets were observed. Subsequently, the sections were immersed in a Cresyl violet staining solution for 2-4 minutes (staining intensity was monitored under a microscope). The sections were washed in multiple changes of distilled water. Dehydration was performed in 70% and two changes of 100% ethanol until no dye cloud was released (3 minutes each). The sections were dehydrated in isopropanol for 3 minutes. Finally, the sections were washed in xylene twice for 3 minutes each before being mounted on slides.

5.7 Immunohistochemistry

For the immunohistochemistry experiments, more than three independent brains were used per group. The AP coordinate for the prefrontal cortex is mPFC (+1.78 mm). Consecutive brain sections were collected, encompassing 5-10 sections before and after the respective AP coordinate for each animal. These sections were specifically utilized for conducting immunofluorescence experiments. Free-floating brain sections were blocked for one hour in a PBS solution containing 3% bovine serum albumin (Sigma Aldrich) and 1% Triton X-100 (Bio-Rad Laboratories). Primary antibodies were applied overnight at 4°C in a PBS solution with 3% bovine serum albumin (Sigma Aldrich). The tissue was washed three times in PBS before incubation for 2 hours with the secondary antibody conjugated to Alexa 555 (red

fluorescence). The brains were subsequently stained with DAPI, and the images were acquired using a confocal microscope (Leica) with a 63× oil objective. The following primary antibodies were utilized in this study: rabbit anti-NFl (1:200; Cell Signaling Technology, 2837), rabbit anti-Iba1 (1:500; Abcam, ab178846), rabbit anti-NeuN (1:500; Abcam, ab177487), rabbit anti-GFAP (1:500; Abcam, ab7260), rabbit anti-BDNF (1:500; Abcam, ab108319), rabbit anti-SOD1 (1:500; proteintech, 10269-1-AP), rabbit anti-Serotonin (1:500; MilliporeSigma, S5545), rabbit anti-JAK2 (phospho Y1007 + Y1008) (1:500; Abcam, A0453), rabbit anti-STAT3 (phospho Y705) (1:500; Abcam, ab76315). In addition, the following secondary antibodies were used: Alexa Fluor 555 donkey anti-rabbit (1:500; Beyotime, A0453).

5.8 In vitro whole-cell patch-clamp recording

Six adult C57/BL6J mice were used for brain slice electrophysiology recordings. Autologous blood was injected into the mPFC, and the mice were dissected 2-4 days later in ice-cold artificial cerebrospinal fluid (ACSF). The ACSF solution contained 125 mM NaCl, 2.5 mM KCl, 1.3 mM $MgSO_4$, 2 mM $CaCl_2$, 26 mM $NaHCO_3$, 1.25 mM NaH_2PO_4, and 10 mM glucose, with a pH of 7.4. Coronal sections of 300 μm containing the mPFC were cut using a vibroslicer, and the sections were allowed to recover in oxygenated ACSF for a minimum of 30 minutes at 35°C. The tissue samples were then transferred to a recording chamber utilizing a submersion-style technique. Oxygenated ACSF solution was continuously superfused at a rate of 4–6 ml/min and maintained at room temperature (20–24°C). The whole-cell patch-clamp technique was used to monitor the membrane voltage, and recording was performed using a MultiClamp 700B patch-clamp amplifier. Patch electrodes were created by pulling thick-walled borosilicate glass capillaries with resistances ranging from 4 to 7 MΩ when filled with an intracellular solution containing 125 mM K^+-gluconate, 0.1 mM $CaCl_2$, 2 mM KCl, 0.3 mM GTP, 4 mM Mg-ATP, 10 mM HEPES, 10 mM EGTA, 8 mM phosphocreatine

sodium, pH 7.2. Neuronal responses were monitored by injecting current pulses (-150, -100, -50, 0, 50, 100, 150 pA), and multiple neurons were recorded in each segment.

5.9 RNA-sequence analysis

Transcriptome sequencing was conducted by Novogene Co., Ltd. on tissue samples obtained from the prefrontal lobe and thalamic hematoma sites. The sequence data for the mPFC and thalamus can be accessed on NCBI GenBank (https://www.ncbi.nlm.nih.gov/) under SRA data: PRJNA1028146. Striatum transcriptome data (GSE216607) was used in the study. The differentially expressed genes (DEGs) between sham and ICH samples were filtered using the DESeq2 method with a padj < 0.05 and $\log_2$ Fold Change >1. Enrichment analysis of the differentially expressed genes based on log2 Fold Change values was performed using the Cluster Profiler package with the Gene Ontology (GO) and Kyoto Encyclopedia of Genes and Genomes (KEGG) databases. The methods for screening depression-related genes: Firstly, differentially expressed genes were identified through GO enrichment and KEGG pathway enrichment analyses based on post-hemorrhagic stroke data. We focused on selecting pathways that have been previously reported and associated with depression, including interferon-gamma (response to interferon-gamma, cellular response to interferon-gamma) [13], antigen processing (antigen processing and presentation, antigen processing and presentation of peptide antigen) [14], regulation of immune effector process [15], and response to interferon-beta [16]. Subsequently, we compiled the genes from the enriched pathways mentioned above. Finally, further screening was conducted based on the following criteria: Condition 1: Gene expression (FPKM values) ratio between the blood group and the control group is greater than 2, with gene expression level (FPKM values) in the blood group greater than 5; Condition 2: Gene expression (FPKM values) ratio between the blood group and the control group is greater than 5, with gene expression level (FPKM values) in the blood group greater than 1. Meeting either of these conditions was considered acceptable.

5.10 RNA extraction and RT-qPCR

RNA extractions were performed using the Trizol method, and cDNA synthesis was carried out using Vazyme's HiScript II 1st Strand cDNA Synthesis Kit (Cat. No.: R2223). Gene mRNA levels were quantified by quantitative reverse transcription-PCR (RT-qPCR) using an ABI 7,500 real-time system. Gapdh expression (NM_001411840) served as the internal control [17]. Control samples from three mice after saline injection on day 7 and mPFC samples from three mice after mPFC-ICH on day 7 were divided into six groups. RNA extracted from each group was considered as one biological replicate, and three replications were analyzed. All primers used are listed in Table S7.

5.11 Administration of inhibitor drug

STAT3-IN-12 is a potent inhibitor of the STAT3 signal and can block the activation of the JAK/STAT3 signaling pathway induced by IL-6 [18]. The dose of STAT3-IN-12 (HY-150538, MedChemExpress) was set at 20 mg/kg, as described previously [18]. In the mPFC-ICH group, STAT3-IN-12 (20 mg/kg) was administered intraperitoneally after ICH, followed by daily administration for 7 days. The control group received an equal volume of 2% DMSO for 7 days. Depression-related behavioral experiments were conducted on day 7 in the four groups, and the expression of JAK2 and STAT3 was measured by immunohistochemistry on day 7 in the four groups.

5.12 Behavioral analysis

Mice undergoing intracerebral hemorrhage surgery underwent pre-operative behavioral testing, followed by post-operative assessments on specific days after inducing ICH models with different volumes (5 μL, 10 μL, and 15 μL). Each brain region was divided into four groups, each containing 6-8 mice. The investigators, who were blinded to the group assignments, recorded and analyzed all behavioral tests. The objective criteria for behavioral evaluation were defined and provided as follows:

Corner turn test: The corner turn test assessed integrated sensorimotor function in mice by stimulating their vibrissae (sensory neglect) and observing their rearing behavior (motor response) [19]. Two pieces of cardboard were arranged to form a 30° angle, and the open end facing the mice was positioned near the corner. The mice gradually approached the corner and reared upward before making a 180° turn to face the open end. The direction (left or right) in which the mouse turned was recorded for each trial. Twelve trials were performed for each mouse.

Basso mouse scale: The Basso mouse scale evaluated the performance of the mice's forelimbs on a rough horizontal pole (8 mm diameter) in three consecutive 30-second tests. The resulting score was as follows [20]: 1 point: The mouse briefly grasped the lower horizontal bar before dropping; 2 point: The mouse grasped and held onto the lower horizontal bar for 11-20 seconds before dropping; 3 point: The mouse grasped and held onto the lower horizontal bar for 21-30 seconds before dropping; 4 point: The mouse grasped and held onto the bottom of the horizontal bar for 30 seconds, but its left hind limb and left forelimb were suspended on the pole; 5-point: The mouse gripped the bottom of the horizontal bar for 30 seconds, but hoisted either the left hind limb or the left forelimb from the pole; 6-point: The mouse tightly held onto the horizontal pole at the bottom for 30 seconds but was unable to sit on it; 7-point: The mouse perched on the pole, but the left hind limb and left forelimb hung down; 8-point: The mouse perched on the pole, but either the left hind or forelimb hung down; 9-point: The mouse moved effortlessly on the pole, and its limbs remained stable.

Beam walking test: The beam walking test involved mice crossing circular wooden beams with a diameter of 1.5 cm, a length of 70 cm, and a height of 30 cm. The corresponding scores were recorded based on walking distance and gait. The test was repeated three times, and the average score was calculated from three consecutive trials. The scoring

system used was as follows, based on a previous study [21]: 0 points: The mouse could not grasp the wooden beam, sat on the wooden beam, or fell directly; 1 point: The mouse could grasp the wooden beam or sit on it, or could not move but can stay for 1 min; 2 points: The mice balanced on the wooden beam or could not pass through the wooden beam but could stay for 1 min; 3 points: The mouse could walk from one end of the beam to the other, but footsteps appear; 4 points: The mice could move freely at one end of the beam to the other.

Modified pole test: The modified pole test assessed mice's movement using a scoring system ranging from 0 to 9 points. The scoring criteria are as follows [22]: 0 points: No ankle exercise; 1 point: Slight ankle movement; 2 points: Extensive ankle mobility; 3 points: Weight support in the plantar position with or without movement; 4 points: Occasional plantar stepping; 5 points: Frequent or consistent plantar steps that are uncoordinated; 6 points: Frequent or consistent plantar steps that are somewhat coordinated, starting with parallel contact of the soles of the feet; 7 point: Frequent or consistent plantar steps, mostly coordinated, with parallel soles of the feet at initial contact and rotation of the soles of the feet during takeoff; 8 point: Frequent or consistent plantar steps, mostly coordinated, with mild trunk instability and parallel soles of the feet at initial contact; 9-point: Regular or consistent plantar steps, mostly coordinated, with parallel soles of the feet at initial contact, normal trunk stability, and a consistently raised tail.

Elevated plus maze: The elevated plus maze (EPM) is commonly used to assess anxiety in laboratory animals. The maze consists of two opposing open and closed arms arranged in a cross formation. The closed arm is enclosed by walls, while the open arm lacks walls. The dimensions of the maze are 30 cm × 5 cm ×15 cm, and it is raised 40 cm above the ground with consistent dim lighting. Mice are placed in the center of the closed arm and allowed to explore the maze for 5 minutes. The placement of the mice is randomized to minimize biases

and preconceptions. Anxiety behavior is quantified using the percentage of time spent in the open arm [23].

Sucrose preference: For the sucrose preference test, the animals were individually housed and provided with two bottles of water for two days, followed by two bottles of a 2% sucrose solution for another two days. Subsequently, the animals were water-deprived for 24 hours and then presented with a bottle of 2% sucrose solution and a bottle of water in the dark phase for two hours. The positions of the bottles were switched after one hour (for the two-hour test). The total sucrose consumption for each liquid was measured, and preference was defined as the average ratio of sucrose consumption, including the first and second hours. The ratio of sucrose consumption is calculated by dividing the total amount of sucrose consumed by the total amount of water and sucrose consumed [23].

Tail suspension: The tail suspension test involved attaching a 17 cm strip of tape marked at the 2 cm point to the tail of the animals, with 2-3 mm of tape overhanging the tail end on the outer side. The taped tail was then suspended from a metal rod. The animals would struggle, and if the struggle did not change their position, they eventually stopped moving. The mice were observed for 6 minutes, and the final 5 minutes were recorded. After completing the experiment, the tape on their tail should be gently removed, and the mice returned to their cage [24].

Forced swim: In the forced swim test, mice are placed in cylindrical glass beakers measuring approximately 10 cm in diameter and 25 cm in height. The beakers are filled with water at approximately 25 °C, with the water level set so that the mice cannot touch the bottom. After a 2-minute habituation period in the water, recording is performed. The duration of immobility is manually scored for four minutes. Following the test, the mice are placed in a pre-warmed cage for 30 minutes before being returned to their home cage [24].

Thermal hyperalgesia: Thermal hyperalgesia experiments were conducted using hot plates. To assess the response time of mice to plantar temperature stimuli on a heated surface, a setup consisting of a shelf plate with thick glass, a heater, and a plexiglass chamber was used. Before testing, the animals were allowed to acclimate indoors for at least 10 minutes. The thermal stimulator was directed towards the surface of the plantar hind paws of the animal and placed under the glass plate. The temperature of the hot plate was set between 52°C to 55°C. The animal's response, in terms of moving away from the heat threshold, indicates delayed response or hyperalgesia. To prevent burn damage, the stimulation duration should not exceed 20 seconds. The process was repeated three to five times, and the average response time was determined as the threshold [25].

Cold hyperalgesia: Thermal hyperalgesia experiments were conducted using hot plates. To assess the response time of mice to plantar temperature stimuli on a cooled surface, a setup consisting of a shelf plate with thick glass, a cooler, and a plexiglass chamber is used Before testing, the animals were allowed to acclimate indoors for at least 10 minutes. The thermal stimulator was directed towards the surface of the plantar hind paws of the animal and placed under the glass plate. The temperature of the hot plate was set between 0°C to 4°C. The animal's response, in terms of moving away from the cold threshold, indicates delayed response or hyperalgesia. To prevent tissue damage, the stimulation duration did not exceed 20 seconds. The process was repeated three to five times, and the average response time was determined as the threshold [26].

5.13 Source attribution for the schematic diagrams

In this study, a portion of the schematic diagrams in the figures was created by imitating and subsequently revising figures from certain references. The references indicate the adoption of a Creative Commons Attribution 4.0 International License for copyright, which allows for the use, sharing, adaptation, distribution, and reproduction of this work in any medium or format,

provided that the original author and source are credited, a link to the Creative Commons license (http://creativecommons.org/licenses/by/4.0/.) is provided, and any modifications made are indicated. Therefore, appreciation is expressed to the following references: Figure 1: The schematic diagrams depicting the mouse model, elevated plus maze, and sucrose preference were created with reference to [27]. Figure 4: The schematic diagram illustrating the whole-brain connectivity of the mPFC was created with reference to [28].

5.14 Statistical analysis

Data in figure legends are presented as mean ± standard deviation (SD) values. The sample size (n) for each statistical analysis is described in the corresponding figure legends. Normality tests were conducted to assess the data distribution, including the Kolmogorov-Smirnov normality test with the Dallal-Wilkinson-Lilliefor P value and Shapiro-Wilk normality test. For normally distributed data, one-way ANOVA was used for multiple group comparisons, and unpaired t-tests (parametric tests) were employed for comparisons between two groups. For non-normally distributed data, a Kruskal-Wallis test followed by Dunn's post hoc test was conducted for multiple group comparisons, and unpaired t-tests (nonparametric tests) were used for comparisons between two groups. All statistical tests were two-tailed, comparing groups of biological replicates. After ANOVA analysis, Tukey's multiple comparison test was performed to determine the p-value between two conditions. Significance values are denoted as follows: Not significant (ns), $P < 0.05$(*), $P < 0.01$(**), and $P < 0.001$(***). Statistical significance tests were performed using GraphPad Prism software (San Diego, CA).

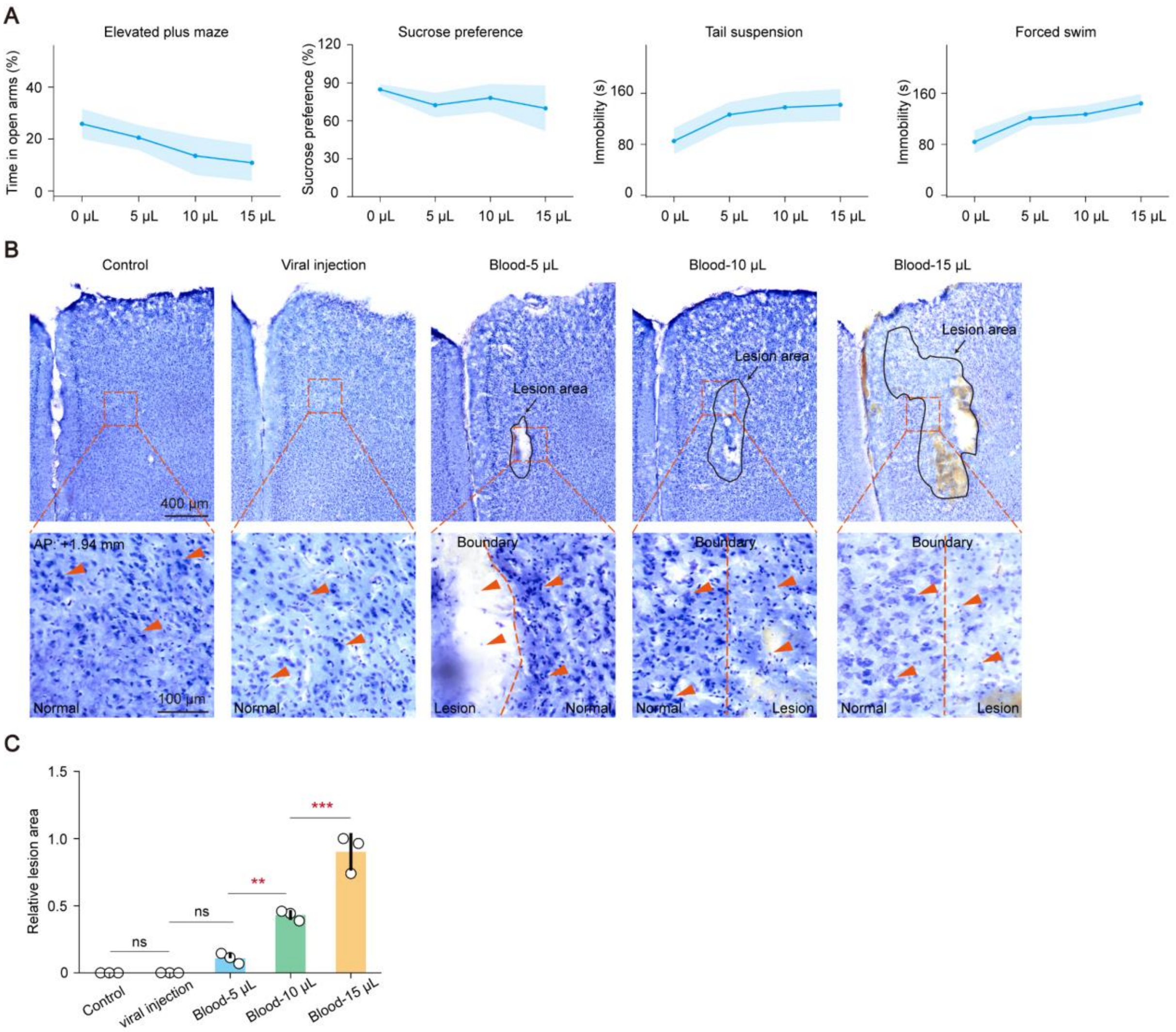

Figure S1. Hematoma in the mPFC region leads to depressive complications in mice. A) Trends of depressive indicators, including performance in the elevated plus maze, sucrose preference, tail suspension, and forced swim tests, were observed on day 7 with increasing hematoma volume. B) CV staining of different groups. In the top row, the black solid line indicates the lesion area, and the red box represents the magnified view. In the bottom row, the red dashed line indicates the boundary between the lesion area and the normal area, and the red arrow points to representative cells. Scale bar: top row, bar = 400 μm; bottom row, bar = 100 μm. C) Statistical analysis of relative lesion area among different groups. Statistical analysis (one-way ANOVA and Tukey's multiple comparison test): $P < 0.0001$, P (Control, viral injection) > 0.9999, P (viral injection, Blood 5 μL) = 0.3395, P (Blood 5 μL, Blood 10

μL) = 0.0012, *P* (Blood 10 μL, Blood 15 μL) < 0.0001. Data are mean ± sd. In (B-C), each group, n = 3 mice. Not significant (ns), *P* < 0.01(**), and *P* < 0.001(***).

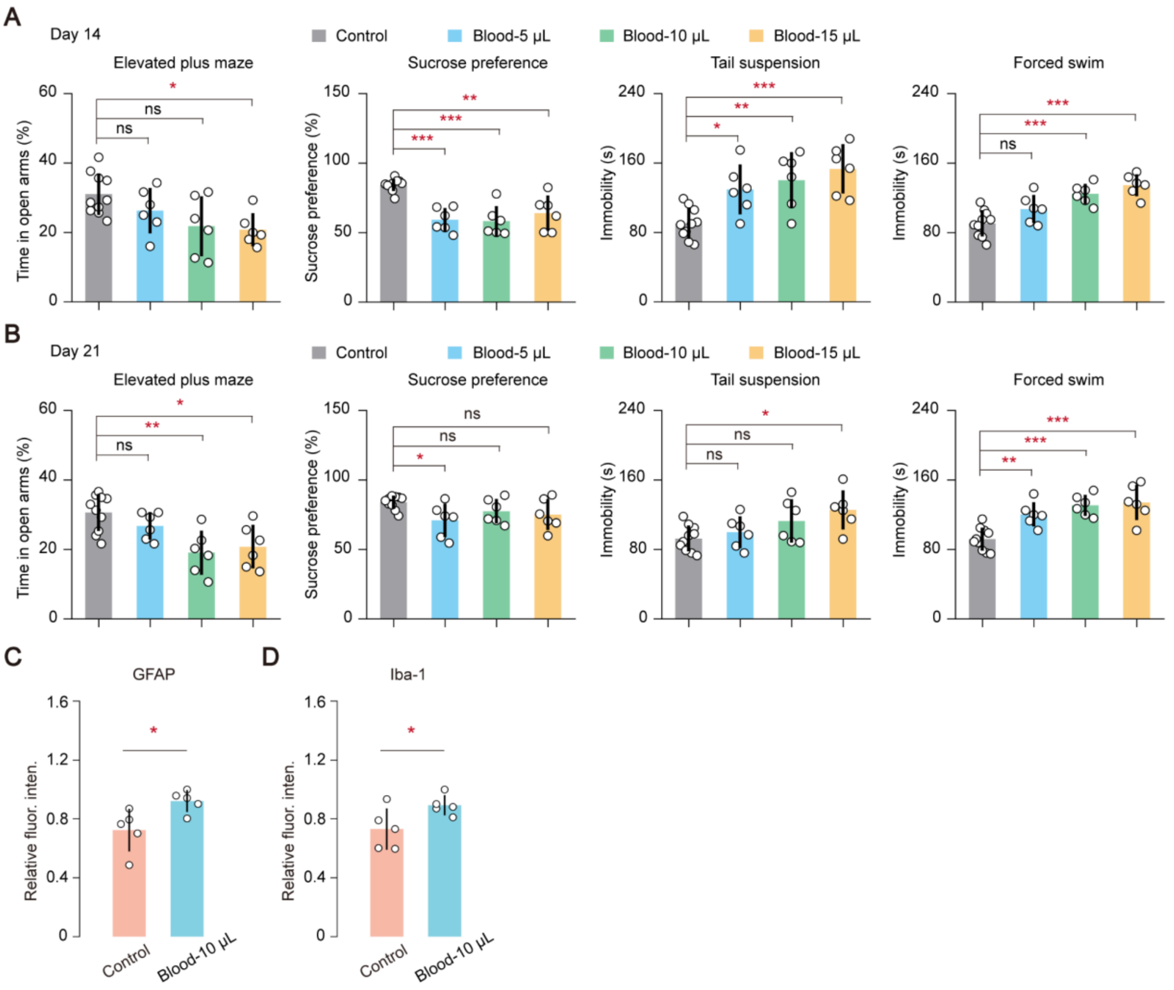

Figure S2. Hematoma in the mPFC region leads to depressive complications in mice.

A) Effect of hematoma volume on elevated plus maze, sucrose preference, tail suspension, and forced swim in different groups on day 14. Statistical analysis (one-way ANOVA and Tukey's multiple comparison test): For elevated plus maze, *P* = 0.0184, *P* (Control, Blood 5 μL) = 0.5119, *P* (Control, Blood 10 μL) = 0.0532, *P* (Control, Blood 15 μL) = 0.0279; for sucrose preference, *P* < 0.0001, *P* (Control, Blood 5 μL) < 0.0001, *P* (Control, Blood 10 μL) < 0.0001, *P* (Control, Blood 15 μL) = 0.0011; for tail suspension, *P* = 0.0004, *P* (Control, Blood 5 μL) = 0.0345, *P* (Control, Blood 10 μL) = 0.0056, *P* (Control, Blood 15 μL) = 0.0005; for forced swim, *P* < 0.0001, *P* (Control, Blood 5 μL) = 0.1364, *P* (Control, Blood 10

μL) = 0.0006, P (Control, Blood 15 μL) < 0.0001; B) Effect of hematoma volume on elevated plus maze, sucrose preference, tail suspension, and forced swim in different groups on day 21. Statistical analysis (one-way ANOVA and Tukey's multiple comparison test): For elevated plus maze, $P = 0.0014$, P (Control, Blood 5 μL) = 0.5470, P (Control, Blood 10 μL) = 0.0025, P (Control, Blood 15 μL) = 0.0107; for sucrose preference, $P = 0.0531$, P (Control, Blood 5 μL) = 0.0437, P (Control, Blood 10 μL) = 0.5146, P (Control, Blood 15 μL) = 0.2456; for tail suspension, $P = 0.0212$, P (Control, Blood 5 μL) = 0.8937, P (Control, Blood 10 μL) = 0.2291, P (Control, Blood 15 μL) = 0.0178; for forced swim, $P < 0.0001$, P (Control, Blood 5 μL) = 0.0058, P (Control, Blood 10 μL) = 0.0002, P (Control, Blood 15 μL) < 0.0001. Data are presented as mean ± sd. C-D) Relative fluorescence density (arbitrary units) of GFAP (C) and Iba-1 (D) near the hematoma. Statistical analysis: Unpaired t-tests (parametric tests). GFAP, P (Control, Blood-10 μL) = 0.0272; Iba-1, P (Control, Blood-10 μL) = 0.0499. Fluorescence density (% area) was analyzed using Default through Image J. Data are mean ± sd. In (A-B), control group, n = 10 mice; blood group, n = 6 mice. In (C-D), each group, n = 5 mice. Not significant (ns), $P < 0.05$(*), $P < 0.01$(**), and $P < 0.001$(***).

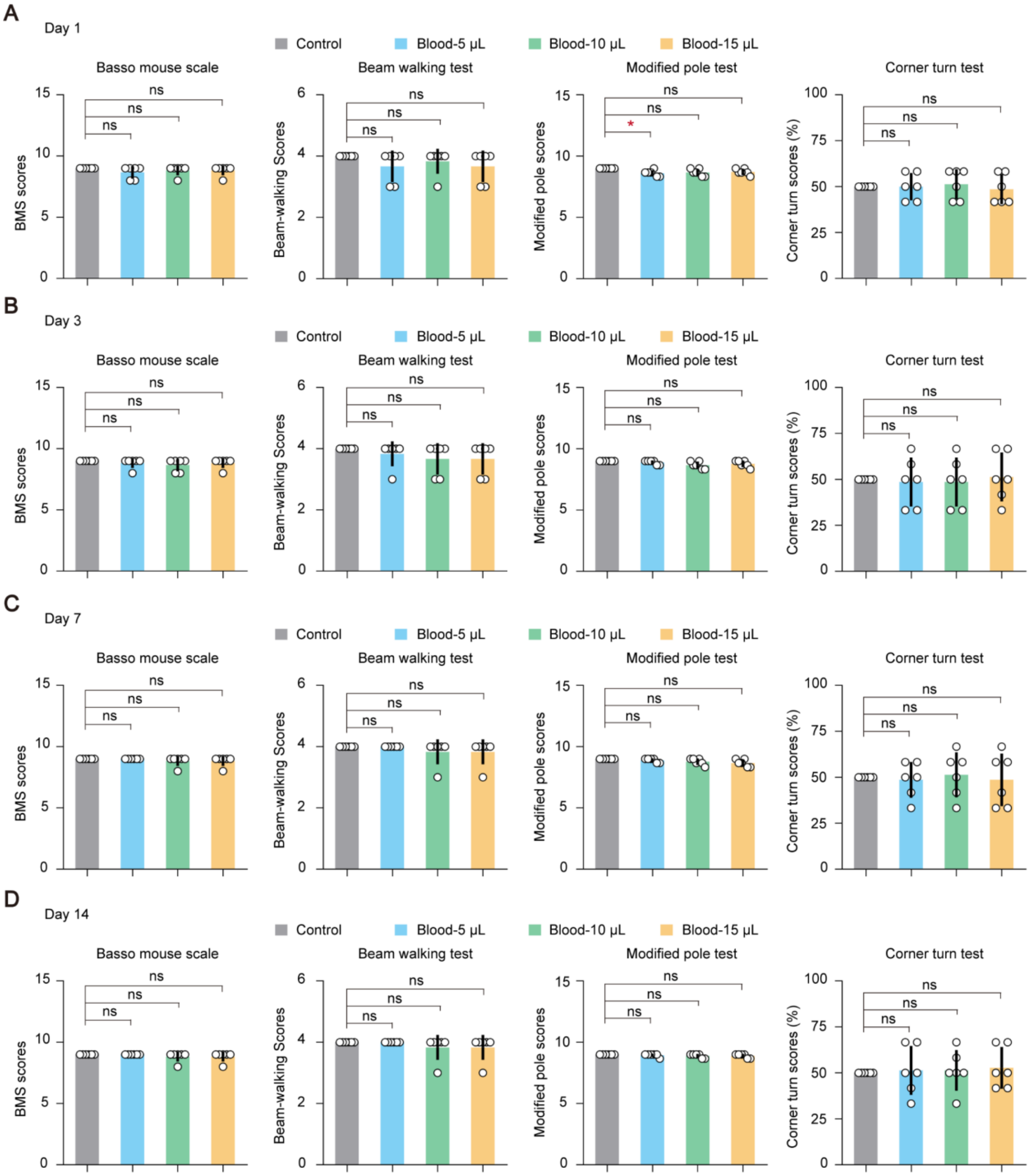

Figure S3. Depression emerges as a specific neurological complication resulting from hematoma in the mPFC region. A-D) Effect of hematoma volume on the basso mouse scale, beam walking test, modified pole test, and corner turn test in different groups on day 1 (A), day 3 (B), day 7 (C), and day 14 (D). Kruskal-Wallis test and Dunn's multiple comparisons test. $P > 0.05$ indicates not significant. Data are mean ± sd. In (A-D), each group, n = 6 mice. Not significant (ns) and $P < 0.05$(*).

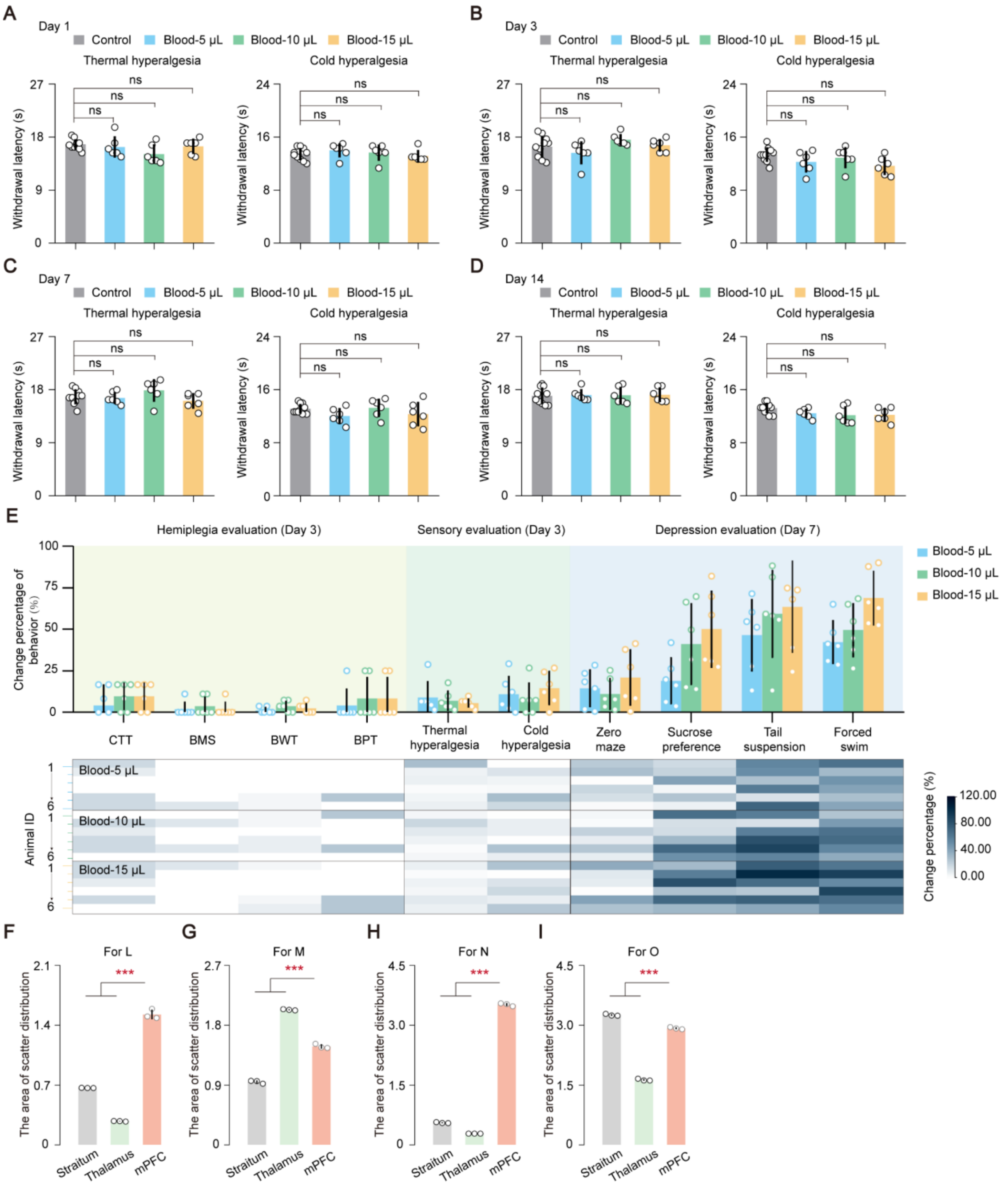

Figure S4. Depression emerges as a specific neurological complication resulting from hematoma in the mPFC region. A-D) Effect of hematoma volume on thermal hyperalgesia and cold hyperalgesia in different groups on day 1 (A), day 3 (B), day 7 (C), and day 14 (D). Kruskal-Wallis test and Dunn's multiple comparisons test. $P > 0.05$ indicates not significant. E) Evaluation of the complications of hemiplegia on day 3, sensory dysfunction on day 3, and depression on day 7 after hematoma in the mPFC. The top panel shows a column chart, and

the bottom panel shows a heat map. Each row in the heat map represents a mouse. The x-axis represents the specific indicator for each behavior, and the y-axis represents the percentage of change of the indicator. F-I) Area of scatter distribution in different blood areas. One-way ANOVA and Tukey’s multiple comparisons test: (F) “Striatum group” = 0.667, “Thalamus group” = 0.278, “mPFC group” = 1.526. $P < 0.0001$, P (Striatum, mPFC) < 0.0001, P (Thalamus, mPFC) < 0.0001; (G) “Striatum group” = 0.949, “Thalamus group” = 2.032, “mPFC group” = 1.481. $P < 0.0001$, P (Striatum, mPFC) < 0.0001, P (Thalamus, mPFC) < 0.0001. (H) “Striatum group” = 0.557, “Thalamus group” = 0.283, “mPFC group” = 3.518. $P < 0.0001$, P (Striatum, mPFC) < 0.0001, P (Thalamus, mPFC) < 0.0001. (I) “Striatum group” = 3.257, “Thalamus group” = 1.631, “mPFC group” = 2.925., $P < 0.0001$, P (Striatum, mPFC) < 0.0001, P (Thalamus, mPFC) < 0.0001. Data are mean ± sd. In (A-D), control group, n = 10 mice; blood group, n = 6 mice. In (E), each group, n = 6 mice. In (F-I), each group, n = 3 mice. Not significant (ns) and $P < 0.001$(***).

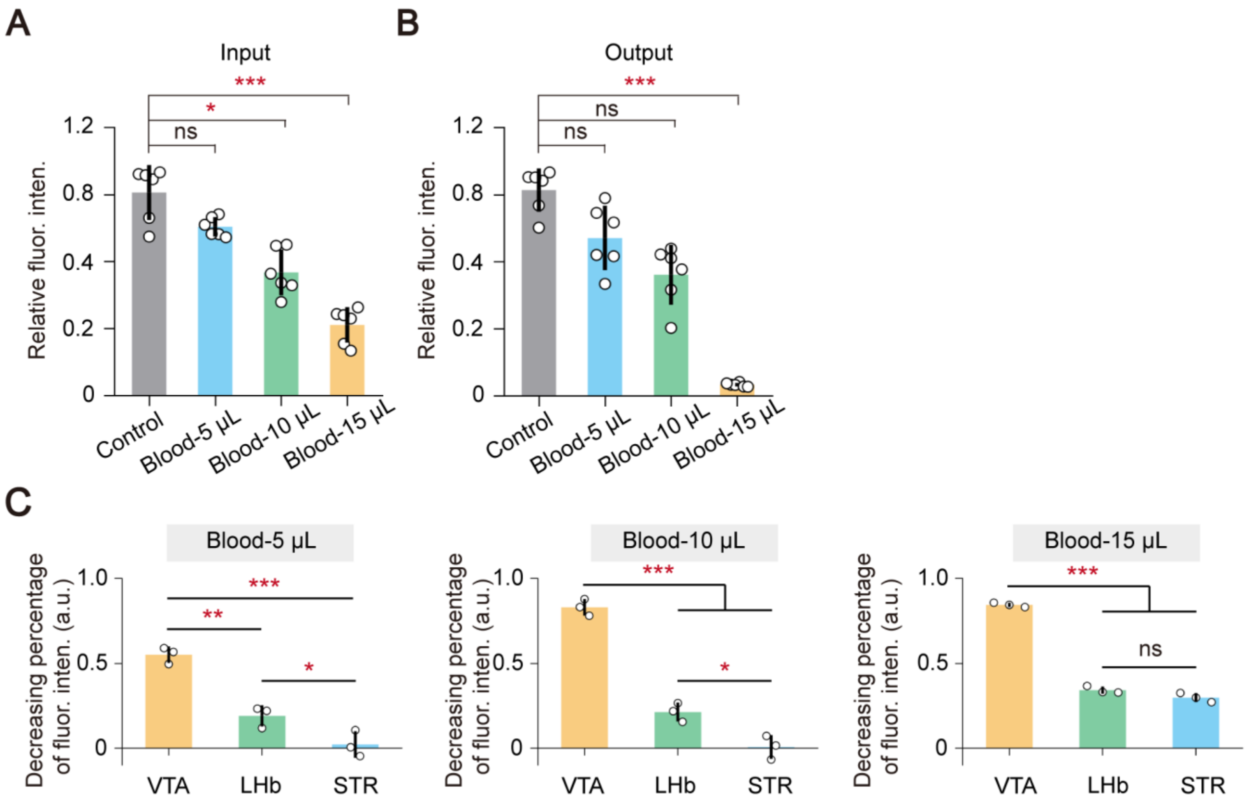

Figure S5. VTA exhibits a higher percentage of connectivity disruption compared to the LHb and STR. A) Relative fluorescence density (arbitrary units) of upstream regions near the hematoma in the four different groups. Statistical analysis: Kruskal-Wallis test, P = 0.0002, Dunn's multiple comparisons test: P (Control, Blood 5 μL) > 0.9999, P (Control, Blood 10 μL) = 0.0478, P (Control, Blood 15 μL) = 0.0002. B) Relative fluorescence density (arbitrary units) of downstream regions near the hematoma in the four different groups. Statistical analysis: Kruskal-Wallis test, P = 0.0003, Dunn's multiple comparisons test: P (Control, Blood 5 μL) = 0.8499, P (Control, Blood 10 μL) = 0.0858, P (Control, Blood 15 μL) = 0.0001. C) Decrease in fluorescence intensity (a.u.) observed in three different areas with varying hematoma volumes. Statistical analysis (one-way ANOVA and Tukey's multiple comparison test): Blood 5 μL, P = 0.0002, P (VTA, LHb) = 0.0012, P (LHb, STR) = 0.0001, P (LHb, STR) = 0.0418; Blood 10 μL, P < 0.0001, P (VTA, LHb) < 0.0001, P (LHb, STR) < 0.0001, P (LHb, STR) = 0.0123; Blood 15 μL, P < 0.0001, P (VTA, LHb) < 0.0001, P (LHb, STR) < 0.0001, P (LHb, STR) = 0.0976. Fluorescence density (% area) was analyzed using

RenyiEntropy through Image J. Data are mean ± sd. In (A-B), each group, n = 6 mice. In (C), each group, n = 3 mice. Not significant (ns), $P < 0.05$(*), $P < 0.01$(**), and $P < 0.001$(***).

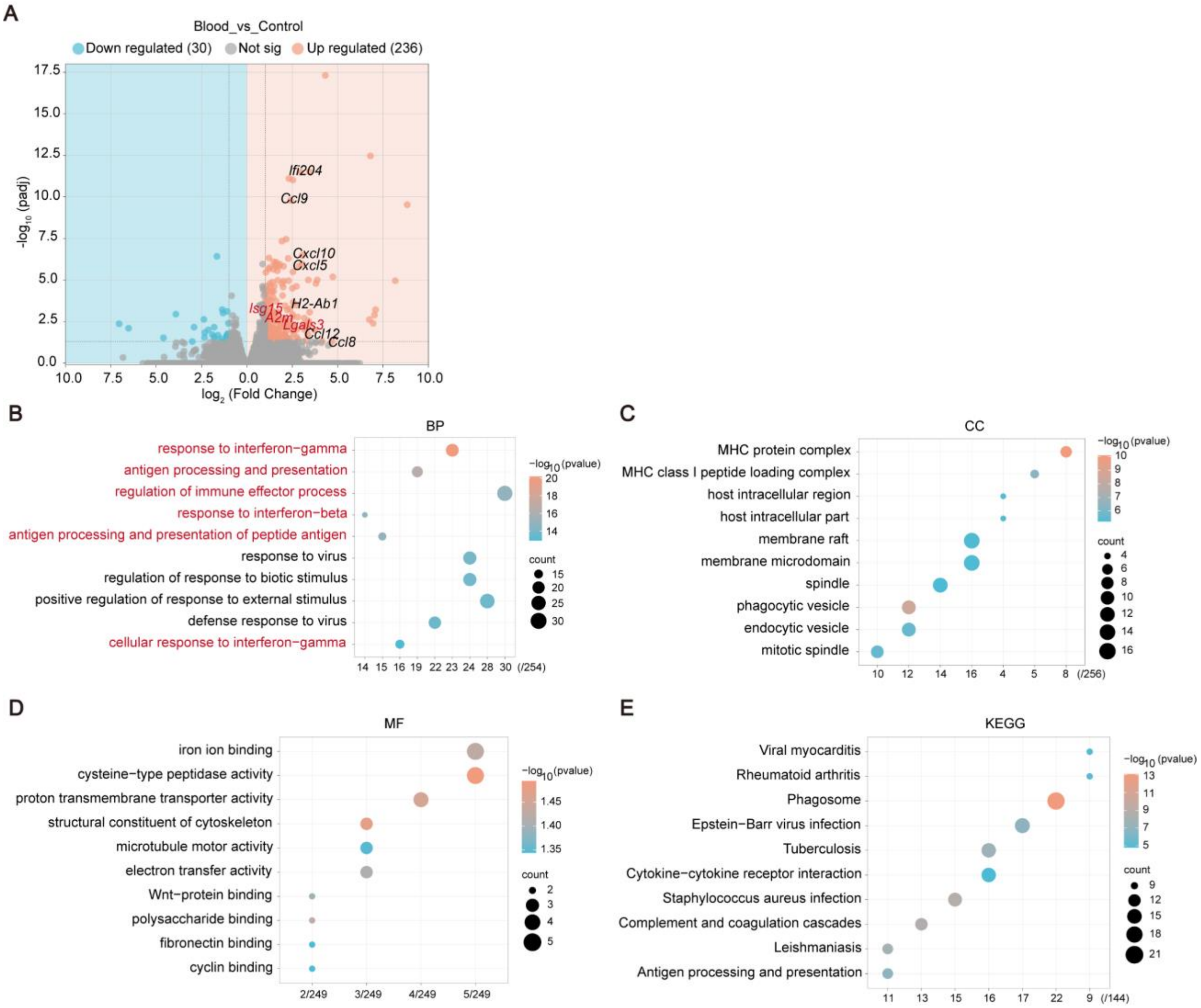

Figure S6. Targeting the JAK-STAT pathway as a potential intervention for depressive symptoms resulting from hematoma in the mPFC. A) DEG in volcano plots comparing the blood groups to the control group. DEG, different expression gene. Blue represents a decrease in difference, gray represents no difference, and pink represents an increase in difference. B-D) Gene Ontology (GO) enrichment analysis of the transcriptome after mPFC-ICH, including Biological Process (BP, B), Cellular Component (CC, C), Molecular Function (MF, D). E) Kyoto Encyclopedia of Genes and Genomes (KEGG) analysis of the transcriptome after mPFC-ICH.

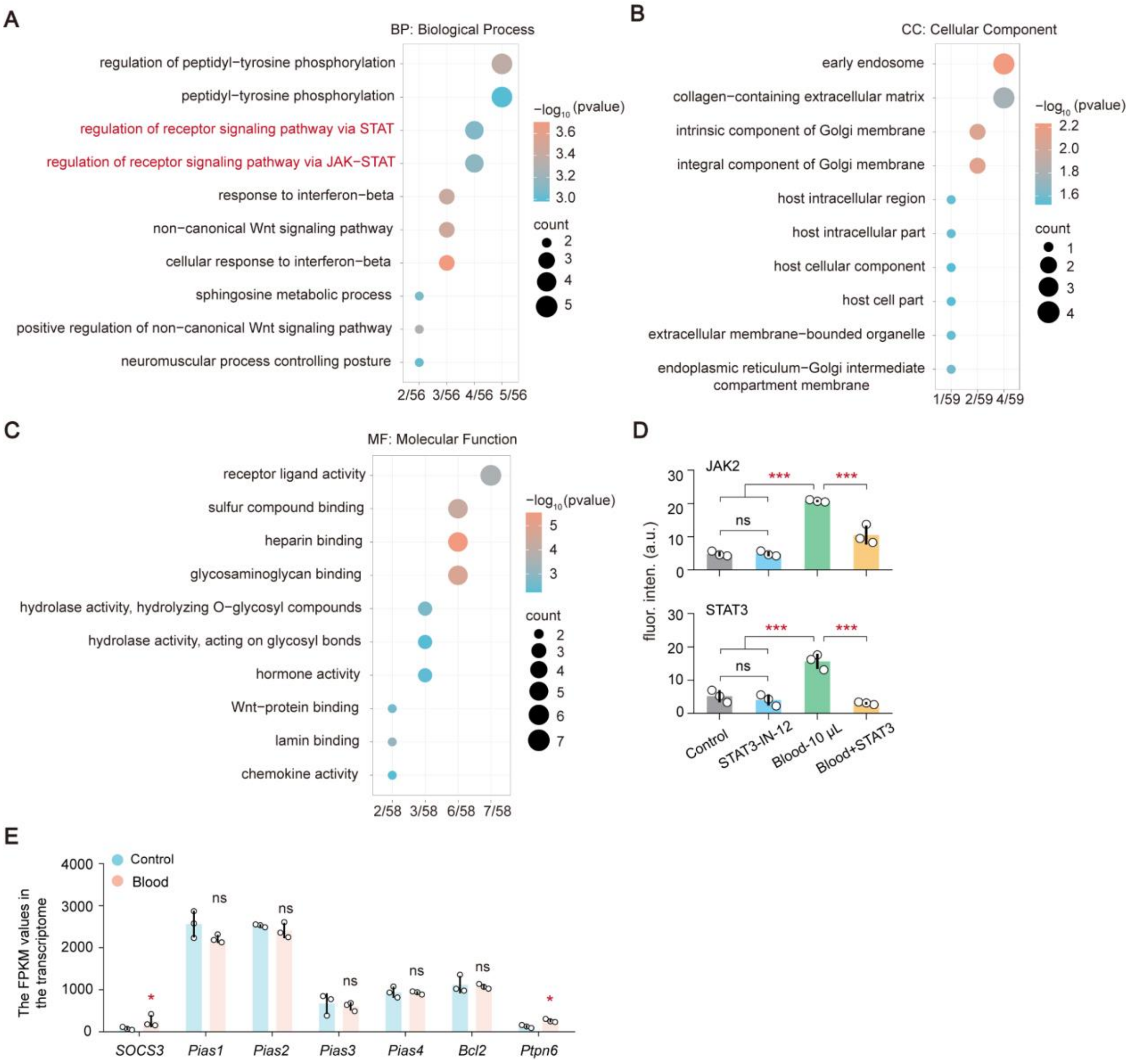

Figure S7. Targeting the JAK-STAT pathway as a potential intervention for depressive symptoms resulting from hematoma in the mPFC. A-C) Analysis of Gene Ontology (GO) enrichment pathways in the 63 specific genes after ICH in the mPFC, linked to biological process (A), cellular component (B), molecular Function (C). D) Quantification of fluorescence intensity of JAK2 and STAT3 in various groups the following results: For JAK2: one-way ANOVA, $P < 0.0001$, Tukey's multiple comparisons test: P (Control, STAT3-IN-12) > 0.9999, P (Control, Blood 10 μL) < 0.0001, P (STAT3-IN-12, Blood 10 μL) < 0.0001, P (Blood 10 μL, Blood + STAT3) = 0.0002; for STAT3: one--way ANOVA, $P <$ 0.0001, Tukey's multiple comparisons test: P (Control, STAT3-IN-12) = 0.8546, P (Control, Blood 10 μL) = 0.0003, P (STAT3-IN-12, Blood 10 μL) = 0.0002, P (Blood 10 μL, Blood +

STAT3) < 0.0001. E) The FPKM values of downstream targets of the JAK-STAT pathway in the transcriptome. *Socs3*: *P* (Control, Blood) = 0.0391; *Pias1*: *P* (Control, Blood) = 0.6282; *Pias2*: *P* (Control, Blood) = 0.8973; *Pias3*: *P* (Control, Blood) = 0.9096; *Pias4*: *P* (Control, Blood) = 0.9826; *Bcl2*: *P* (Control, Blood) = 0.9494; *Ptpn6*: *P* (Control, Blood) = 0.0245. Data are mean ± sd. In (D-E), each group, n = 3 mice. Not significant (ns), $P < 0.05$(*), and $P < 0.001$(***).

Table S1.

Significant statistical differences for each time point in Figure 1G.

Table S2.

Genes with differential expression in the medial prefrontal cortex (mPFC) compared to the control group in blood samples.

Table S3.

Genes related to depression with differential expression in the control and blood groups.

Table S4.

Specific differential expression genes in the mPFC were obtained by comparing three different transcriptome datasets.

Table S5.

Gene Ontology (GO) enrichment pathways in 63 specific genes after hematoma in the mPFC, including Biological Process (BP), Cellular Component (CC), and Molecular Function (MF).

Table S6.

Kyoto Encyclopedia of Genes and Genomes (KEGG) enrichment pathways in 63 specific genes after hematoma in the mPFC.

Table S7.

Primer information for key genes related to depression after hematoma in the mPFC.

References

[1] a) L. Qian, S. Huang, X. Liu, Y. Jiang, Y. Jiang, Y. Hu, Z. Yang, *Phytomedicine* **2024**, *123*, 155224, https://doi.org/10.1016/j.phymed.2023.155224; b) X. Ke, M. Deng, Z. Wu, H. Yu, D. Yu, H. Li, Y. Lu, K. Shu, L. Pei, *Neurosci Bull* **2023**, *39* (2), 194, https://doi.org/10.1007/s12264-022-00898-7; c) F. Qinlin, X. Qi, C. Qiong, X. Lexing, S. Peixia, H. Linlin, D. Yupu, Y. Lijun, Y. Qingwu, *Bioengineered* **2022**, *13* (2), 3582, https://doi.org/10.1080/21655979.2022.2027061.

[2] a) Y. C. Liao, Y. C. Hu, C. P. Chung, Y. F. Wang, Y. C. Guo, Y. S. Tsai, Y. C. Lee, *Stroke* **2021**, *52* (3), 985, https://doi.org/10.1161/STROKEAHA.120.030664; b) A. C. Leasure, A. I. Qureshi, S. B. Murthy, H. Kamel, J. N. Goldstein, K. B. Walsh, D. Woo, F. D. Shi, H. B. Huttner, W. C. Ziai, D. F. Hanley, C. C. Matouk, L. H. Sansing, G. J. Falcone, K. N. Sheth, *Stroke* **2019**, *50* (8), 2016, https://doi.org/10.1161/STROKEAHA.119.024838; c) D. J. Seiffge, S. Curtze, N. Dequatre-Ponchelle, A. Pezzini, T. Tatlisumak, C. Cordonnier, D. Werring, *Neurology* **2019**, *92* (8), e782, https://doi.org/10.1212/WNL.0000000000006958.

[3] A. Etkin, T. Egner, R. Kalisch, *Trends Cogn Sci* **2011**, *15* (2), 85, https://doi.org/10.1016/j.tics.2010.11.004.

[4] a) J. L. Price, W. C. Drevets, *Trends Cogn Sci* **2012**, *16* (1), 61, https://doi.org/10.1016/j.tics.2011.12.011; b) N. Eshel, G. C. Touponse, A. R. Wang, A. K. Osterman, A. N. Shank, A. M. Groome, L. Taniguchi, D. F. Cardozo Pinto, J. Tucciarone, B. S. Bentzley, R. C. Malenka, *Neuron* **2024**, *112* (3), 500, https://doi.org/10.1016/j.neuron.2023.10.038.

[5] E. De Falco, M. Solca, F. Bernasconi, M. Babo-Rebelo, N. Young, F. Sammartino, C. Tallon-Baudry, V. Navarro, A. R. Rezai, V. Krishna, O. Blanke, *Proc Natl Acad Sci U S A* **2024**, *121* (11), e2316365121, https://doi.org/10.1073/pnas.2316365121.

[6] J. Xu, Z. Chen, F. Yu, H. Liu, C. Ma, D. Xie, X. Hu, R. K. Leak, S. H. Y. Chou, R. A. Stetler, Y. Shi, J. Chen, M. V. L. Bennett, G. Chen, *Proc Natl Acad Sci U S A* **2020**, *117* (51), 32679, https://doi.org/10.1073/pnas.2018497117.

[7] D. Klebe, L. Iniaghe, S. Burchell, C. Reis, O. Akyol, J. Tang, J. H. Zhang, *Methods Mol Biol* **2018**, *1717*, 83, https://doi.org/10.1007/978-1-4939-7526-6_7.

[8] a) J. Li, R. Zhao, C. Wang, J. Song, X. Guo, Y. Ge, X. Chu, *Pain Rep* **2024**, *9* (2), e1139, https://doi.org/10.1097/PR9.0000000000001139; b) X. Wang, B. Cheng, N. Roberts, S. Wang, Y. Luo, F. Tian, S. Yue, *Hum Brain Mapp* **2021**, *42* (16), 5458, https://doi.org/10.1002/hbm.25618; c) T. B. Meier, W. C. Drevets, B. E. Wurfel, B. N. Ford, H. M. Morris, T. A. Victor, J. Bodurka, T. K. Teague, R. Dantzer, J. Savitz, *Brain Behav Immun* **2016**, *53*, 39, https://doi.org/10.1016/j.bbi.2015.11.003.

[9] a) L. Wang, M. Gao, Q. Wang, L. Sun, M. Younus, S. Ma, C. Liu, L. Shi, Y. Lu, B. Zhou, S. Sun, G. Chen, J. Li, Q. Zhang, F. Zhu, C. Wang, Z. Zhou, *Nat Commun* **2023**, *14* (1),

1568, https://doi.org/10.1038/s41467-023-37045-3; b) D. Liu, Q. Q. Tang, C. Yin, Y. Song, Y. Liu, J. X. Yang, H. Liu, Y. M. Zhang, S. Y. Wu, Y. Song, B. Juarez, H. L. Ding, M. H. Han, H. Zhang, J. L. Cao, *Pain* **2018**, *159* (1), 175, https://doi.org/10.1097/j.pain.0000000000001083; c) S. Lin, L. Huang, Z. C. Luo, X. Li, S. Y. Jin, Z. J. Du, D. Y. Wu, W. C. Xiong, L. Huang, Z. Y. Luo, Y. L. Song, Q. Wang, X. W. Liu, R. J. Ma, M. L. Wang, C. R. Ren, J. M. Yang, T. M. Gao, *Biol Psychiatry* **2022**, *92* (3), 179, https://doi.org/10.1016/j.biopsych.2022.02.014; d) J. Liu, J. W. Mo, X. Wang, Z. An, S. Zhang, C. Y. Zhang, P. Yi, A. T. L. Leong, J. Ren, L. Y. Chen, R. Mo, Y. Xie, Q. Feng, W. Chen, T. M. Gao, E. X. Wu, Y. Feng, X. Cao, *Sci Adv* **2022**, *8* (46), eabo2098, https://doi.org/10.1126/sciadv.abo2098.

[10] F. Wang, C. B. Liu, Y. Wang, X. X. Wang, Y. Y. Yang, C. Y. Jiang, Q. M. Le, X. Liu, L. Ma, F. F. Wang, *Theranostics* **2024**, *14* (3), 1126, https://doi.org/10.7150/thno.90792.

[11] S. Ma, M. Chen, Y. Jiang, X. Xiang, S. Wang, Z. Wu, S. Li, Y. Cui, J. Wang, Y. Zhu, Y. Zhang, H. Ma, S. Duan, H. Li, Y. Yang, C. J. Lingle, H. Hu, *Nature* **2023**, *622* (7984), 802, https://doi.org/10.1038/s41586-023-06624-1.

[12] B. N. Phan, M. H. Ray, X. Xue, C. Fu, R. J. Fenster, S. J. Kohut, J. Bergman, S. N. Haber, K. M. McCullough, M. K. Fish, J. R. Glausier, Q. Su, A. E. Tipton, D. A. Lewis, Z. Freyberg, G. C. Tseng, S. J. Russek, Y. Alekseyev, K. J. Ressler, M. L. Seney, A. R. Pfenning, R. W. Logan, *Nat Commun* **2024**, *15* (1), 878, https://doi.org/10.1038/s41467-024-45165-7.

[13] M. Maes, S. Scharpe, H. Y. Meltzer, G. Okayli, E. Bosmans, P. D'Hondt, B. V. Vanden Bossche, P. Cosyns, *Psychiatry Res* **1994**, *54* (2), 143, https://doi.org/10.1016/0165-1781(94)90003-5.

[14] X. Shen, D. Caramaschi, M. J. Adams, R. M. Walker, J. L. Min, A. Kwong, G. Hemani, D. N. A. M. C. Genetics of, M. C. Barbu, H. C. Whalley, S. E. Harris, I. J. Deary, S. W. Morris, S. R. Cox, C. L. Relton, R. E. Marioni, K. L. Evans, A. M. McIntosh, *Genome Med* **2022**, *14* (1), 36, https://doi.org/10.1186/s13073-022-01039-5.

[15] L. E. Santos, D. Beckman, S. T. Ferreira, *Brain Behav Immun* **2016**, *55*, 151, https://doi.org/10.1016/j.bbi.2015.11.011.

[16] A. Feinstein, P. O'Connor, K. Feinstein, *J Neurol* **2002**, *249* (7), 815, https://doi.org/10.1007/s00415-002-0725-0.

[17] N. L. Cook, T. J. Kleinig, C. van den Heuvel, R. Vink, *BMC Mol Biol* **2010**, *11*, 7, https://doi.org/10.1186/1471-2199-11-7.

[18] Y. C. Liu, Y. D. Yang, W. Q. Liu, T. T. Du, R. Wang, M. Ji, B. B. Yang, L. Li, X. G. Chen, *Bioorg Med Chem* **2022**, *65*, 116757, https://doi.org/10.1016/j.bmc.2022.116757.

[19] S. S. Karuppagounder, I. Alim, S. J. Khim, M. W. Bourassa, S. F. Sleiman, R. John, C. C. Thinnes, T. L. Yeh, M. Demetriades, S. Neitemeier, D. Cruz, I. Gazaryan, D. W. Killilea, L. Morgenstern, G. Xi, R. F. Keep, T. Schallert, R. V. Tappero, J. Zhong, S. Cho, F. R. Maxfield, T. R. Holman, C. Culmsee, G. H. Fong, Y. Su, G. L. Ming, H. Song, J. W. Cave, C. J. Schofield, F. Colbourne, G. Coppola, R. R. Ratan, *Sci Transl Med* **2016**, *8* (328), 328ra29, https://doi.org/10.1126/scitranslmed.aac6008.

[20] S. Schneider, A. Gruart, S. Grade, Y. Zhang, S. Kroger, F. Kirchhoff, G. Eichele, J. M. Delgado Garcia, L. Dimou, *Glia* **2016**, *64* (12), 2201, https://doi.org/10.1002/glia.23055.

[21] W. Wu, W. Xiong, P. Zhang, L. Chen, J. Fang, C. Shields, X. M. Xu, X. Jin, *PLoS One* **2017**, *12* (5), e0178803, https://doi.org/10.1371/journal.pone.0178803.

[22] K. Socala, D. Nieoczym, M. Pierog, E. Wyska, M. Szafarz, U. Doboszewska, P. Wlaz, *Neurotox Res* **2018**, *34* (3), 333, https://doi.org/10.1007/s12640-018-9876-4.

[23] M. Li, C. Li, H. Yu, X. Cai, X. Shen, X. Sun, J. Wang, Y. Zhang, C. Wang, *J Neuroinflammation* **2017**, *14* (1), 190, https://doi.org/10.1186/s12974-017-0964-9.

[24] Z. Fan, J. Chang, Y. Liang, H. Zhu, C. Zhang, D. Zheng, J. Wang, Y. Xu, Q. J. Li, H. Hu, *Cell* **2023**, *186* (3), 560, https://doi.org/10.1016/j.cell.2022.12.033.
[25] V. Viatchenko-Karpinski, L. Kong, H. R. Weng, *Glia* **2022**, *70* (4), 634, https://doi.org/10.1002/glia.24130.
[26] C. Wang, X. Jin, Q. Zhang, H. Wang, H. Ji, Y. Zhou, C. Zhu, Y. Yang, G. Yu, Z. Tang, *Br J Anaesth* **2023**, https://doi.org/10.1016/j.bja.2023.08.010.
[27] H. Yu, L. Chen, H. Lei, G. Pi, R. Xiong, T. Jiang, D. Wu, F. Sun, Y. Gao, Y. Li, W. Peng, B. Huang, G. Song, X. Wang, J. Lv, Z. Jin, D. Ke, Y. Yang, J. Z. Wang, *Nat Commun* **2022**, *13* (1), 5462, https://doi.org/10.1038/s41467-022-33139-6.
[28] P. G. Anastasiades, A. G. Carter, *Trends Neurosci* **2021**, *44* (7), 550, https://doi.org/10.1016/j.tins.2021.03.006.
